# Vitamin D, Folate, and Cobalamin Serum Concentrations Are Related to Brain Volume and White Matter Integrity in Urban Adults

**DOI:** 10.3389/fnagi.2020.00140

**Published:** 2020-05-25

**Authors:** May A. Beydoun, Danielle Shaked, Sharmin Hossain, Hind A. Beydoun, Leslie I. Katzel, Christos Davatzikos, Rao P. Gullapalli, Stephen L. Seliger, Guray Erus, Michele K. Evans, Alan B. Zonderman, Shari R. Waldstein

**Affiliations:** ^1^Laboratory of Epidemiology and Population Sciences, The National Institute on Aging (NIA) The Intramural Research Program (IRP), The National Institutes of Health (NIH), Baltimore, MD, United States; ^2^Department of Psychology, University of Maryland, Baltimore County, MD, United States; ^3^Department of Research Programs, Fort Belvoir Community Hospital, Fort Belvoir, VA, United States; ^4^Geriatric Research Education and Clinical Center, Baltimore VA Medical Center, Baltimore, MD, United States; ^5^Division of Gerontology & Geriatric Medicine, Department of Medicine, University of Maryland School of Medicine, Baltimore, MD, United States; ^6^Section for Biomedical Image Analysis, Department of Radiology, University of Pennsylvania, Philadelphia, PA, United States; ^7^Department of Diagnostic Radiology, University of Maryland School of Medicine, Baltimore, MD, United States; ^8^Division of Nephrology, Department of Medicine, University of Maryland School of Medicine, Baltimore, MD, United States

**Keywords:** 25-hydroxyvitamin D, folate, cobalamin, brain volumes, white matter integrity, cognitive aging, health disparities

## Abstract

**Background and objectives:** Lower vitamin status has been linked to cognitive deficits, pending mechanistic elucidation. Serum 25-hydroxyvitamin D [25(OH)D], folate and cobalamin were explored against brain volumes and white matter integrity (WMI).

**Methods:** Two prospective waves from Healthy Aging in Neighborhoods of Diversity Across the Life Span (HANDLS) study were primarily used [Baltimore, City, MD, 2004–2015, *N* = 183–240 urban adults (Age_v1_: 30–64 years)]. Serum vitamin 25-hydroxyvitamin D [25(OH)D], folate and cobalamin concentrations were measured at visits 1 (v_1_: 2004–2009), while structural and diffusion Magnetic Resonance Imaging (sMRI/dMRI) outcomes were measured at vscan: 2011–2015. Top 10 ranked adjusted associations were corrected for multiple testing using familywise Bonferroni (FWER < 0.05) and false discovery rates (FDR, *q*-value < 0.10).

**Results:** We found statistically significant (FWER < 0.05; β±SE) direct associations of 25(OH)D(v_1_) with WM volumes [overall: +910 ± 336/males: +2,054 ± 599], occipital WM; [overall: +140 ± 40, males: +261 ± 67 and Age_v1_ > 50 years: +205 ± 54]; parietal WM; [overall: +251 ± 77, males: +486 ± 129 and Age_v1_ > 50 years: +393 ± 108] and left occipital pole volume [overall: +15.70 ± 3.83 and above poverty: 19.0 ± 4.3]. Only trends were detected for cobalamin exposures (*q* < 0.10), while serum folate (v_1_) was associated with lower mean diffusivity (MD) in the Anterior Limb of the Internal Capsule (ALIC), reflecting greater WMI, overall, while regional FA (e.g., cingulum gyrus) was associated with greater 25(OH)D concentration.

**Conclusions:** Among urban adults, serum 25(OH)D status was consistently linked to larger occipital and parietal WM volumes and greater region-specific WMI. Pending longitudinal replication of our findings, randomized controlled trials of vitamin D supplementation should be conducted against brain marker outcomes.

## Introduction

A possible beneficial effect of several vitamins on cognition has been suggested (Beydoun et al., 2014a). Vitamin D is a steroid hormone that regulates calcium homeostasis. Serum 25-hyrdoxyvitamin D [25(OH)D], or vitamin D status, is primarily determined by sunlight skin exposure and secondarily by dietary and supplemental intakes (Buell and Dawson-Hughes, 2008). Vitamin D's active form (1,25-dihydroxyvitamin D_3_) maintains and stabilizes intracellular signaling pathways involved in memory and cognition (Eyles et al., 2013) by increasing *VDR* (Guo et al., 2016) and *LRP2* expression in the choroid plexus and helping clear neurotoxic β-amyloids (Deane et al., 2004; Carro et al., 2005) involved in Alzheimer's disease (AD) pathogenesis (Roher et al., 1993). Vitamin D-related gene polymorphisms and lower vitamin D intake and status were linked to cognitive decline in epidemiological studies (Annweiler et al., 2016; Kuzma et al., 2016; Beydoun et al., 2018; Goodwill et al., 2018) and to markers of brain atrophy and poor white matter integrity (WMI) (Buell et al., 2010; Annweiler et al., 2013, 2015b; Michos et al., 2014; Prager et al., 2014; Brouwer-Brolsma et al., 2015; Del Brutto et al., 2015; Moon et al., 2015; Karakis et al., 2016; Littlejohns et al., 2016; Al-Amin et al., 2019). Vitamin D's neuroprotective role is likely mediated through the expression of neurotrophins, neurotransmitters, and suppression of inflammatory cytokines (Buell and Dawson-Hughes, 2008; Miller, 2010; Etgen et al., 2012).

Moreover, folate and cobalamin (vitamin B-12) are essential in remethylation of homocysteine (Hcy), a sulfur amino acid with neurotoxic and excitotoxic properties (Kruman et al., 2000), yielding methionine (Bottiglieri, 2005; Troesch et al., 2016). Hcy was recently shown in animal studies to increase tau protein phosphorylation, truncation, and oligomerization, an evidence of direct involvement in AD's second pathological hallmark, namely neurofibrillary tangles (NFTs) (Shirafuji et al., 2018). Hcy is also converted to cysteine via a vitamin B6-dependent mechanism (Troesch et al., 2016). Importantly, folate and cobalamin status were inversely associated with age-related cognitive decline (McCaddon and Miller, 2015; Smith and Refsum, 2016), with cobalamin further exhibiting direct associations with brain volumes and WMI (Erickson et al., 2008; Vogiatzoglou et al., 2008; De Lau et al., 2009; Pieters et al., 2009; Lee et al., 2016). A recent trial demonstrated beneficial effects of B-vitamin supplementation on brain magnetic resonance imaging (MRI) measures and cognitive function longitudinally (De Jager et al., 2012; Douaud et al., 2013). Furthermore, nutritional biomarkers may work synergistically to improve cognitive outcomes (Min and Min, 2016; Moretti et al., 2017). Since socio-demographic factors relate to both nutrition and cognition (Beydoun et al., 2014b; Berg et al., 2015; McCarrey et al., 2016; Weuve et al., 2018), studying relations of vitamin D, folate and cobalamin with brain MRI measures, while stratifying by relevant sociodemographic factors is key.

This study examines associations of serum 25(OH)D, folate and cobalamin concentrations with brain volume and WMI among a diverse sample of urban adults, while stratifying by sex, age, race, and poverty status. We hypothesized that first-visit serum 25(OH)D, folate, and cobalamin (and annual rate of change over time) would directly correlate with global and regional gray and white matter (WM and GM) brain volumes and regional WMI measured at one follow-up visit (v_scan_), after a mean follow-up of 5.7 years. Analyses also explored brain regions' sensitivity to lower vitamin status, differentially by socio-demographic factors.

## Methods and Materials

### Database

Using area probability sampling, a socio-demographically diverse sample of middle-aged White and African-American urban adults (Age v_1_: 30–64 years) from thirteen contiguous census tracts of Baltimore was recruited into the Healthy Aging of Neighborhoods of Diversity across the Life Span (HANDLS) study (Evans et al., 2010). HANDLS is an on-going prospective cohort study, initiated in 2004 by the National Institute on Aging. Potential participants were interviewed and identified by random selections of address listings within each census tract (Evans et al., 2010). Participants were invited to join HANDLS if they met the following criteria: (1) between ages 30–64; (2) not currently pregnant; (3) not within 6 months of active cancer treatment; (4) not diagnosed with AIDS; (5) capable of providing written informed consent, thus excluding individuals with probable dementia or very low literacy among others; (6) with a valid government-issued identification and a verifiable address (Evans et al., 2010).

Initial examinations were performed in two phases. Phase 1 included the first dietary interview and completion of various demographic and psychosocial scales. Phase 2, performed on Medical Research Vehicles (MRV) parked in participants' neighborhoods, included the second dietary interview and various physical, medical, and psychosocial examinations, including DXA for bone mineral density and body composition, EKG, intima-media thickness by ultrasound, personal and family health history, physical examination by a physician, physical performance by a brief screening battery, neuropsychological tests, and inventories to assess depressive symptoms (Evans et al., 2010). Follow-up visits included largely comparable MRV visits. At visit 2 (v_2_, 2009–2013), blood draw analyzed in the same laboratory facility as for visit 1 yielded similar biochemical and hematological indices that can be studied longitudinally.

Written informed consent was obtained from all participants. Study protocols for HANDLS and HANDLS SCAN were approved by the National Institute on Environmental Health Sciences Institutional Review Board (IRB) of the National Institutes of Health. HANDLS SCAN was also approved by the IRBs of the University of Maryland School of Medicine and the University of Maryland, Baltimore County.

This study analyzed nutritional biomarker data from visit 1 (v_1_: 2004–2009) in relation to follow-up data measured in a sub-sample of N_max_ = 258 participants within the HANDLS SCAN sub-study (vscan: 2011–2015) (Waldstein et al., 2017). Exposure variables were measured during the Medical Research Vehicle (MRV) examinations; outcomes were MRI measures of brain volume and WMI at vscan (Waldstein et al., 2017). The mean follow-up time between visit 1 and vscan was 5.70 years ± 1.90.

### Study Sample

**Figure 1:**
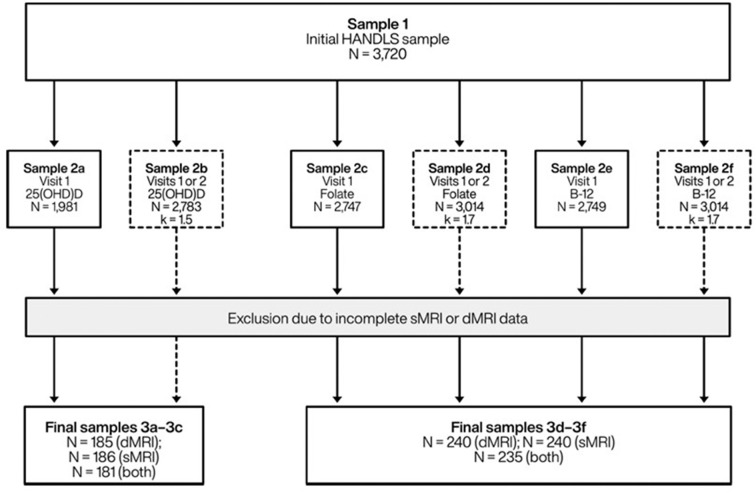
Study participant schematic: HANDLS 2004–2013 and HANDLS-SCAN 2011–2015^a^. 25(OH)D, 25-hydroxyvitamin D; B-12, Vitamin B-12 (cobalamin); dMRI, Diffusion magnetic resonance imaging; HANDLS, Healthy Aging in Neighborhoods of Diversity Across the Life Span; k, average number of repeats; sMRI, Structural/volumetric magnetic resonance imaging. ^a^Visit 1 refers to HANDLS 2004–2009; Visit 2 refers to HANDLS 2009–2013; and HANDLS-SCAN visit (v_scan_) was carried out between 2011 and 2015.

The initial HANDLS cohort included 3,720 participants (30–65 years, AAs and Whites, Phase I, visit 1). We included participants with complete and valid MRI data at follow-up and complete 25(OH)D, folate and cobalamin data at visit 1 and/or visit 2 (Figure 1). Mean ± SD of follow-up time between v_1_ and v_2_ was 4.65 years ± 0.93 (range: 0.4–8.2 years). The final sample was reduced to *N* = 185–186 for vitamin D and *N* = 240 for folate or cobalamin exposures.

### Brain sMRI

A Siemens Tim-Trio 3.0 Tesla scanner was used for MRI assessments. Magnetization prepared rapid gradient echo (MP-RAGE) was used to perform volumetric measurements for anatomical regions. Volumetric measures were estimated for each region of interest (ROI). Detailed description is provided in Supplemental Method 1.

In addition to standard axial T1, T2, FLAIR images, a high-resolution axial T1-weighted MPRAGE (TE = 2.32 ms, TR = 1900 ms, TI = 900 ms, flip angle = 9°, resolution = 256 × 256 × 96, FOV = 230 mm, sl. Thick. = 0.9 mm) of the brain was acquired for structural imaging. Images were used as anatomic references and to extract parameters of regional and whole brain volumes (see Supplemental Table 1). This study comprehensively examines brain volumes at ascending segmentation levels.

### Brain dMRI

dMRI was obtained using multi-band spin echo EPI sequence with a multi-band acceleration factor of three (Supplemental Method 1). Fractional Anisotropy (FA) and trace (TR, aka mean diffusivity or MD) images were computed from tensor images. As intact WM generally allows for more restricted diffusion, higher FA values are indicative of healthier WMI. Summing eigenvalues for diffusion tensor yields MD, with higher values indicative of poorer WMI (Jones, 2008). Computed FA and MD images were aligned to a common template space via deformable registration using a standard dMRI template (i.e., EVE Wakana et al., 2004). Right and left FA and MD values were averaged for each ROI (see Supplemental Table 2 for list of ROIs).

### Vitamin Status Measures

Participants were asked to fast for ≥8 h prior to the MRV visits, and specimens in volumes of 2 mL serum were collected and frozen at −80°C. Similar procedures were adopted for v_1_ and v_2_ serum folate and cobalamin, measured using chemiluminescence immunoassay^1^ by Quest Diagnostics, Chantilly, VA^2^, and previously validated against other automated methods with coefficient of variation (CV) < 10% (Owen and Roberts, 2003; Ispir et al., 2015).

25(OH)D were measured using slightly different methodologies between v_1_ and v_2_. For both visits, blood samples drawn at examination were stored at −80°C. At v_1_, total levels of serum 25(OH)D (in ng/ml; D_2_ and D_3_) were measured using tandem mass spectrometry (interassay CV, 8.6%) at Massachusetts General Hospital, < 60 days later, as recommended for frozen samples (Powe et al., 2013). V_2_ 25(OH)D was measured by Quest Diagnostics (Chantilly, VA) using an immunoassay that includes competitive binding of serum 25(OH)D and tracer-labeled 25(OH)D to specific antibody followed by detection and quantitation via chemiluminescence reaction (Diasorin, formerly Incstar), comparable to National Health and Nutrition Examination Surveys 2003–04 assays^3^ (interassay CV: 4–13%) (Centers for Disease Control Prevention, 2006; Diagnostics, 2019)^4^.

Dietary and supplemental intakes of vitamin D, folate and cobalamin were shown to moderately correlate with their corresponding serum biomarkers in HANDLS and national surveys (Beydoun et al., 2010a,b, 2018). Moreover, moderate-to-strong correlations were detected for all three biomarkers (Pearson's *r* > 0.30), notably v_1_ vs. v_2_ values for each vitamin in the HANDLS sample: 25(OH)D (*r* = 0.44, *n* = 1,462); folate (*r* = 0.44, *n* = 1,944); cobalamin (*r* = 0.55, *n* = 1,994). We also describe categorical exposures with cutoffs reflecting vitamin insufficiency or deficiency (Snow, 1999; Thacher and Clarke, 2011; World Health Organization, 2015).

### Covariates

All models were adjusted for baseline examination age (y), sex (male = 1, female = 0), race (AA = 1, White = 0), self-reported household income either < 125% or ≥125% of the 2004 Health and Human Services poverty guidelines (termed poverty status) (US Department of Health & Human Services, 2019), and time (days) between baseline MRV visit and MRI scan visit. Models were independently stratified by age ( ≤ 50 vs. >50 years), sex, race, or poverty status. Additional covariates were entered in a sensitivity analysis when independently associated with each exposure of interest (see Supplemental Method 2).

### Statistical Analysis

Analyses were conducted using Stata version 16.0 (Stata, 2019). First, selected sample characteristics were described, and their means and proportions across key socio-demographic groups were calculated. *T*-test, chi-square, multiple linear, and logistic regression models (Wald tests) were used to determine group differences in distributions of continuous and categorical variables. Second, several sets of analyses were conducted to test main hypotheses, both overall and stratified by age group (≤ 50 vs. >50 years), sex, race, or poverty status. Ordinary least square regression models included each v_1_ vitamin exposure predicting each MRI measure as the outcome measured at v_scan_, while adjusting for socio-demographic confounders. Ultimately, the most significant adjusted associations with the lowest *p*-values [or highest –Log_10_(p)] per analysis were selected, along with their unstandardized (β±SE) and standardized (*b*) effect sizes. Consequently, a looping procedure (*parmsby* command) was applied to generate main parameter estimates, interpreted as change in MRI measure per unit change in serum vitamin biomarker for β and fraction of a SD change in MRI measure per 1 SD change in that biomarker for *b*, which was moderate-to-strong if >0.20, and weak-to-moderate if between 0.10 and 0.20. Four separate analyses were conducted based on MRI variable groupings. The first analysis included total brain volume (i.e., WM + GM), WM and GM volumes as the only 3 exposure measures (**Model A**). The second analysis included 8 measures (**Model B**): the combination of WM and GM of the 4 main cortical regions: frontal, temporal, parietal, and occipital lobes. A third analysis included the smaller regions, accounting for bilateral volumes, yielding 142 outcome measures (**Model C**). Finally, dMRI measures were included, after taking the average between the left and right side for FA and MD measures, as done previously (McKay et al., 2019). This exploratory approach was conducted previously by at least one other study of vitamin D deficiency and WMI (Moon et al., 2015). This resulted in 98 (49 FA and 49 MD) dMRI outcome measures, reflecting WMI (**Model D**).

For uncorrected *p*-values, Type I error < 0.05 was used for significance. To adjust for multiple testing two methods were used: (1) Familywise Bonferroni (error rate) correction (FWER) which adjusted for multiplicity in brain MRI measures, assuming each set of modeling approach (Models A-D and stratification status) applied to each serum vitamin [25(OH)D, folate and cobalamin] to be separate hypotheses, (2) false discovery rate (*q*-value) which only considered the four approaches/stratification status as separate hypotheses (i.e., Models A-D, and stratification status), thus combining the 3 vitamin exposures upon correction. Moreover, the top 10 adjusted associations from each analysis were presented if *p*_uncorr_ < 0.05, showing the main parameter estimate and its standard error (SE), the uncorrected *p*-values, the FDR *q*-values and FWER status (Yes = passed correction, No = did not pass) and the standardized effect size *b*. Top 10 associations were considered statistically significant if passing FWER correction for a specific vitamin, model and stratification status (yes vs. no) at type I error of 0.05. Results with FDR *q*-value < 0.10 per model and stratification status while failing the FWER criterion were considered a trend. Additionally, when passing FDR *q*-value correction at type I error of 0.10 per vitamin, model and stratification status while failing the FWER criterion, an effect was considered a trend if |*b*| ≥0.20. Among selected stratified models (top 10 findings), formal effect modification testing was conducted by including 2-way interaction terms between exposure and each socio-demographic factor in the non-stratified model. A Type I error of 0.10 was used for 2-way interaction terms due to reduced statistical power (Selvin, 2004). In addition, the main analyses with v_1_ exposures and minimal socio-demographic adjustment, sensitivity analyses were conducted with additional adjustments (Supplemental Method 2).

Using R version 3.6.1, selected findings for Model D, were presented using volcano plots (R Foundation for Statistical Computing, 2013). These plots display Log_10_(*p*-values) for each set of models against *b* on the X-axis, highlighting findings with larger *b*. For dMRI results, these plots were presented separately for FA and MD, given their expected inverse correlation. Visualization of ROI-specific *b* with standard brain images was carried out using FSLeyes software (Jenkinson and Smith, 2001; Jenkinson et al., 2002) applied to dMRI results (URL: https://fsl.fmrib.ox.ac.uk/fsl/fslwiki/FSLeyes). Only ROIs with uncorrected *p*-value < 0.05 are presented.

## Results

Greater serum concentrations of 25(OH)D and folate were observed among Whites relative to AAs, with the reverse pattern observed for cobalamin. All three serum concentrations were consistently higher among “above poverty” participants (vs. below poverty), while only 25(OH)D and folate were higher in those aged >50 years at v_1_ (vs. ≤ 50 years). Larger total and regional volumes among males, Whites, and those living above poverty (for total and GM volume) were detected compared to their counterparts (*p* < 0.05). The older group had smaller frontal GM volumes than the younger group, and differences by poverty status were mostly notable for occipital and frontal volumes (GM and WM). After multivariable adjustment, most poverty status differences in volumes became non-significant. For simplicity, only larger ROIs are presented (Table 1).

**Table 1:** Study sample characteristics by sex, age group, race and poverty status; HANDLS 2004–2009 and HANDLS-SCAN 2011–2015^a^.

	**Total**	**Females**	**Males**	**≤50 years**	**>50 years**	**White**	**African-American**	**Below poverty**	**Above poverty**
	**(*****N*** **=** **240)**	**(*****N*** **=** **135)**	**(*****N*** **=** **105)**	**(*****N*** **=** **143)**	**(*****N*** **=** **97)**	**(*****N*** **=** **141)**	**(*****N*** **=** **99)**	**(*****N*** **=** **79)**	**(*****N*** **=** **161)**
**DEMOGRAPHIC FACTORS**									
Sex, % males	41.3	__	__	41.3	47.4	44.0	43.4	35.4	47.8
Age_v1_	47.7 ± 8.9	47.7 ± 0.8	47.9 ± 0.8	41.6 ± 0.5^c^	56.7 ± 0.4	49.0 ± 0.7^c^^,^^d^	46.1 ± 1.0	44.3 ± 0.9^c^	49.3 ± 0.7
Race, % AA	41.2	41.5	41.0	46.9^b^^,^^d^	32.0	__	__	56.6^c^^,^^d^	74.5
% above poverty	67.1	62.2	73.3	57.3^c^	81.4	74.5^3^	56.6	__	__
	**(*****N*** **=** **183)**	**(*****N*** **=** **99)**	**(*****N*** **=** **84)**	**(*****N*** **=** **105)**	**(*****N*** **=** **78)**	**(*****N*** **=** **108)**	**(*****N*** **=** **75)**	**(*****N*** **=** **54)**	**(*****N*** **=** **129)**
**VITAMIN STATUS (v**_**1**_**)**									
25(OH)D, *ng/mL*	22.3 ± 10.8	20.9 ± 1.1	23.9 ± 1.1	20.5 ± 1.0^c^^,^^d^	24.7 ± 1.2	26.7 ± 1.0^c^	15.9 ± 0.9	17.2 ± 1.4^c^	24.4 ± 0.9
Median	20.0	19.0	23.0	19.0	22.5	25.5	15.0	15.5	23.0
IQR	14.0–31.0	12.0–30.0	16–32.5	12–29	16–33	19.0–34.5	10.0–19.0	9.0–21.0	16.0–33.0
% < 20	37.1	40.7	32,4	39.2	34.0	20.6^c^	60.6	48.1^c^^,^^d^	31.7
% < 10	9.6	12.6	5.7	14.0^c^^,^^d^	3.1	2.8^c^	19.2	20.3^c^	4.4
	**(*****N*** **=** **240)**	**(*****N*** **=** **135)**	**(*****N*** **=** **105)**	**(*****N*** **=** **143)**	**(*****N*** **=** **97)**	**(*****N*** **=** **141)**	**(*****N*** **=** **99)**	**(*****N*** **=** **79)**	**(*****N*** **=** **161)**
Serum folate, ng/mL	15.0 ± 6.3	15.0 ± 0.6	15.0 ± 0.6	13.5 ± 0.5^c^	17.4 ± 0.6	16.0 ± 0.5^c^^,^^d^	13.6 ± 0.6	13.1 ± 0.6^c^	16.1 ± 0.5
Median	14.3	14.7	14.2	12.6	17.9	15.4	12.7	12.5	15.2
IQR	9.5–20.6	9.2–20.6	9.5–20.5	8.9–17.1	12.2–22.5	10.6–21.3	8.5–17.5	8.6–17.1	10.6–21.3
% < 6	6.3	7.4	4.8	8.4	3.1	4.3	9.1	7.6	5.6
Serum B-12, *pg/mL*	518.7 ± 239.7	535.4 ± 23.0	497.2 ± 19.3	502.7 ± 18.3	542.3 ± 27.1	488.0 ± 19.7^b^	562.6 ± 24.3	475.3 ± 19.2^b^	540.0 ± 20.9
Median	463.0	464.0	456.0	460.0	464.0	438.0	521.0	455	467.0
IQR	360.0–626.5	374.0–631.0	347–623.0	359–571	362.0–644.0	339.0–571.0	390.0–679.0	338.0–457.0	364.0–644.0
% < 200	0.42	__	__	__	__	__	__	__	__
**BRAIN VOLUMES (v**_**scan**_**)**, ***mm**^**3**^*									
Total brain volume	970,454 ± 104,344	921,280 ± 6,311^c^	1,033,677 ± 10,198	978,724 ± 8,702	958,261 ± 10,569	989,978 ± 8,947^c^	942,645 ± 9,587	951,587 ± 11,157^b^^,^^d^	979,711 ± 8,343
Gray matter	513,545 ± 5,6152	488,776 ± 3,542^c^	545,391 ± 5,558	519,446 ± 4,752	504,846 ± 5,510	526,576 ± 4,657^c^	494,985 ± 5,249	502,325 ± 6,015^b^^,^^d^	518,559 ± 4,485
White matter	456,908 ± 51,582	432,504 ± 3,111^c^	488,286 ± 5,036	459,278 ± 4,214	453,414 ± 5,417	463,402 ± 4,531^b^	447,660 ± 4,720	448,261 ± 5,506	461,151 ± 4,134
Gray matter: Frontal	179,001 ± 20,690	170,642 ± 1,350^c^	189,748 ± 2,092	181,228 ± 1,788^b^	175,421 ± 1,947	183,082 ± 1,772^c^	173,188 ± 1890	175,430 ± 2,166^b^^,^^d^	180,752 ± 1,671
Gray matter: temporal	98,813 ± 11,598	93,343 ± 740^c^	105,847 ± 1,091	99,454 ± 982	97,869 ± 1,154	101,081 ± 956^c^	95,584 ± 1,128	96,870 ± 1255	99,767 ± 924
Gray matter: occipital	68,691 ± 9,035	65,145 ± 607^c^	73,251 ± 907	69,202 ± 765	67,937 ± 900	71,392 ± 730^c^	64,846 ± 817	66,637 ± 962^c^^,^^d^	69,699 ± 718
Gray matter: parietal	87,585 ± 11,259	83,786 ± 787^c^	92,470 ± 1,158	88,950 ± 930^b^	85,572 ± 1138	90,446 ± 926^c^	83,510 ± 1,044	85,916 ± 1,243	88,404 ± 891
White matter: Frontal	186,294 ± 21,618	176,870 ± 1,353^c^	198,412 ± 2,164	187,094 ± 1,791	185,115 ± 2,230	188,256 ± 1,888	183,500 ± 2,031	182,321 ± 2,275^b^^,^^d^	188,243 ± 1,739
White matter: temporal	104,302 ± 12,020	98,399 ± 688^c^	111,893 ± 1,181	104,782 ± 969^e^	103,596 ± 1,284	106,1044 ± 1,050^c^	101,750 ± 1,107	102,559 ± 1,268	105,158 ± 970
White matter: occipital	45,860 ± 6,113	43,155 ± 414^c^	49,338 ± 571	46,394 ± 509^e^	45,073 ± 619	46,879 ± 538^c^	44,410 ± 543	44,775 ± 627^b^^,^^d^	46,392 ± 497
White matter: parietal	90,621 ± 11,436	85,721 ± 765^c^	96,920 ± 1,101	91,074 ± 939	89,951 ± 1,193	92,253 ± 1,018^b^	88,295 ± 1,009	89,171 ± 1,270	91,332 ± 904

*25(OH)D, 25-hydroxivitamin D; Age_v1_, age measured at HANDLS visit 1 (2004–2009); HANDLS, Healthy Aging in Neighborhoods of Diversity Across the Life Span; HANDLS-SCAN, Brain magnetic resonance imaging scan ancillary study of HANDLS; IQR, Interquartile range (25th−75th percentile); v_1_, visit 1 of HANDLS (2004–2009); v_scan_, HANDLS-SCAN visit (2011–2015)*.

a *Values are Mean ± SD for totals and Mean ± SE for stratum-specific, or %. For 25(OH)D, folate and vitamin B-12, medians and inter-quartile ranges (IQR) were also provided. N = 183 for 25(OH)D analysis. The sample is that of HANDLS participants with complete visit 1 folate/B-12 measures and sMRI data [N = 240 for most analysis; N = 183 for 25(OH)D]. See methods for cutoffs chosen for each vitamin. Cobalamin deficiency analysis yielded only 1 participant below the 200 pg/mL cutoff. Thus, stratified analysis was not conducted*.

b *P < 0.05*.

c *P < 0.010 for null hypothesis of no difference by sex, age group, race, or poverty status, t-test (continuous variables), and chi-squared test (categorical variables)*.

d *P ≥ 0.05 after adjustment for remaining covariates, multiple linear regression (continuous variables), multiple logistic regression (categorical variables)*.

e *P < 0.05 after adjustment for remaining covariates, multiple linear regression (continuous variables), multiple logistic regression (categorical variables)*.

**Figure 2:**
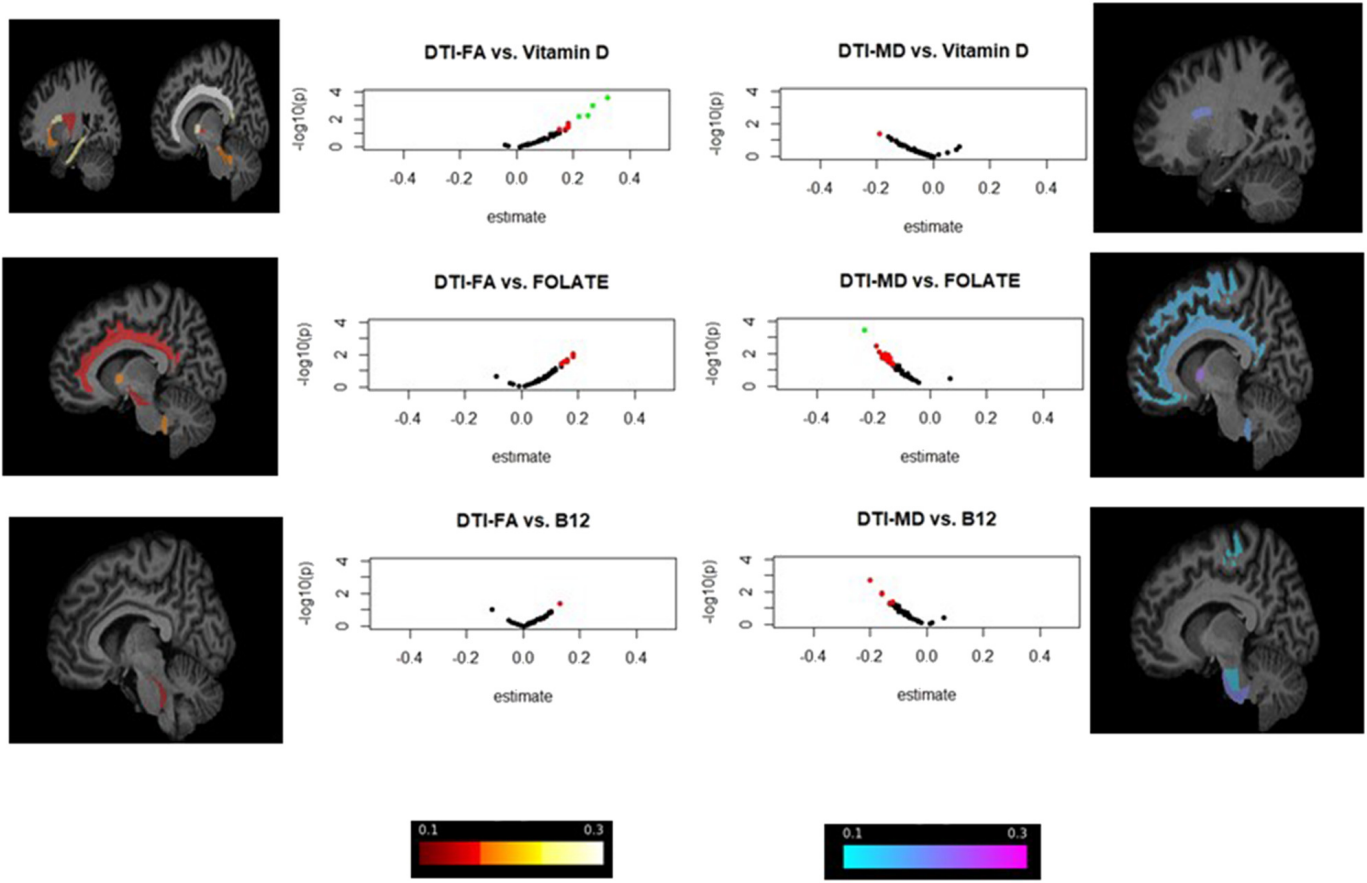
ROI-wide brain dMRI association with v_1_ serum 25(OH)D, folate and cobalamin in total population: volcano plots and brain image visualization for HANDLS 2004–2009 and HANDLS-SCAN 2011–2015^a,b^. ^a^Volcano plots display Log_10_(*p*-values) for each set of models against the standardized effect (b) on the X-axis, highlighting findings with larger effect sizes. Associations with *P* < 0.05 are presented in red, whereas those with both *P* < 0.05 and effect size in absolute value >0.20 are presented in green. ^b^Brain visualization using FSLeyes program is focused on standardized effect sizes (b) and direction, with negative effects (*b* < 0) shown in cold colors and positive effects (*b* > 0) shown in warmer colors. The range is between −0.3 and +0.3 with lighter colors indicating stronger effects in either direction. Only ROIs with uncorrected *p*-value < 0.05 are presented.

Top 10 adjusted associations with uncorrected *p* < 0.05 from ordinary least square brain scan-wide analyses are presented in Tables 2–**4** and Figure 2. Among significant findings (FWER < 0.05) in the main analysis (Table 2), serum 25(OH)D was directly associated with larger WM volumes [overall (β = +910 ± 336, *p* = 0.007, *q* = 0.067, passed FW Bonferroni correction), effect size *b* = 0.19], with a stronger effect size among men (*b* = 0.41). This association was specific to occipital and parietal WM, with a moderate effect size (*b* = +0.23–0.25, *q* < 0.05, passed FW Bonferroni correction) in the overall sample, men and the older group. A trend toward a direct association was also detected between 25(OH)D and total brain volume in the overall sample, in men and those in the older group. Among trends (*q*-value < 0.10), temporal and occipital WM volumes were directly associated with 25(OH)D, in Whites and individuals living above poverty, respectively. Most of these 25(OH)D vs. larger ROIs associations were not altered when additional covariates were entered in a sensitivity analysis (Table 2). Higher cobalamin exhibited a trend association with larger total brain, total GM, frontal and occipital GM volumes in the overall sample (*q*-value < 0.10), becoming null after adjustment for 25(OH)D and other covariates (see Supplemental Method 2).

**Table 2:** Top 10 adjusted associations from models A (total, GM, WM) and B (regional GM, WM) vs. visit 1 exposures: serum 25(OH)D, folate and cobalamin (overall and stratified analysis) with uncorrected *P* < 0.05: ordinary least square brain scan-wide analyses on HANDLS 2004–2009 and HANDLS-SCAN 2011–2015^a^.

	**Outcome (v_**scan**_)**	**Outcome description**	**Exposure (v_**1**_)**	**Stratum**	**(*N*)**	**β**	**(*SE*)**	***P*_**uncorr**_**	**Standardized Beta (b)**	***q*-value**	**Passes FW Bonferroni correction**	**Standardized Beta (b): SA^b^**	***P*_**uncorr**_: SA**
**MODEL A**													
	**WM**	**White matter**	**25(OH)D**	**__**	**(186)**	**+910**	**(336)**	**0.007**	**+0.19**	**0.067**	**Yes**	**+0.18**	**0.017**
Overall	TOTALBRAIN	Total brain volume	25(OH)D	__	(186)	+1554	(659)	0.019	+0.16	0.087^d^	No	+0.15	0.033
**Stratified**	**WM**	**White matter**	**25(OH)D**	**Males**	**(87)**	**+2054**^c^	**(599)**	**0.001**	**+0.41**	**0.069**	**Yes**	**+0.43**	**0.002**
	WM	White matter	25(OH)D	>50 years	(80)	+1500^c^	(470)	0.002	+0.31	0.076^d^	No	+0.25	0.017
	TOTALBRAIN	Total brain volume	25(OH)D	Males	(87)	+3537^c^	(1180)	0.004	+0.34	0.087^d^	No	+0.38	0.005
	TOTALBRAIN	Total brain volume	25(OH)D	>50 years	(80)	+2551^c^	(891)	0.005	+0.28	0.098^d^	No	+0.22	0.023
	GM	Gray matter	25(OH)D	Males	(87)	+1481	(630)	0.021	+0.26	0.29	No	+0.30	0.022
	WM	White matter	25(OH)D	AP	(132)	+930	(406)	0.024	+0.18	0.29	No	***+0.16***	***0.088***
	GM	Gray matter	25(OH)D	>50 years	(80)	+1051	(471)	0.029	+0.22	0.29	No	***+0.19***	***0.052***
	GM	Gray matter	B-12	AP	(161)	+28	(13)	0.034	+0.13	0.29	No	+0.08	0.26
	TOTALBRAIN	Total brain volume	25(OH)D	AP	(132)	+1663	(789)	0.037	+0.16	0.29	No	***+0.15***	***0.085***
	TOTALBRAIN	Total brain volume	B-12	AP	(161)	52	(26)	0.044	+0.13	0.29	No	+0.07	0.31
**MODEL B**													
**Overall**	**OCCIPITAL_WM**	**Occipital white matter**	**25(OH)D**	**__**	**(186)**	**+140**	**(40)**	**5.2e-04**	**+0.25**	**0.012**	**Yes**	**+0.24**	**0.001**
	**PARIETAL_WM**	**Parietal white matter**	**25(OH)D**	**__**	**(186)**	**+251**	**(77)**	**1.5e-03**	**+0.23**	**0.017**	**Yes**	**+0.22**	**0.004**
	PARIETAL_GM	Parietal gray matter	25(OH)D	__	(186)	+191	(74.9)	1.2e-02	+0.18	0.086^d^	No	+0.18	0.016
	FRONTAL_GM	Frontal gray matter	B-12	__	(240)	+11.2	(5)	1.6e-02	+0.13	0.086^d^	No	+0.07	0.27
	OCCIPITAL_GM	Occipital gray matter	B-12	__	(240)	+4.8	(2.0)	1.8e-02	+0.13	0.086^d^	No	+0.10	0.12
	TEMPORAL_WM	Temporal white matter	25(OH)D	__	(186)	+178	(77)	2.2e-02	+0.16	0.089^d^	No	+0.15	0.039
	FRONTAL_WM	Frontal white matter	25(OH)D	__	(186)	+309	(149)	3.9e-02	+0.15	0.13	No	+0.13	0.079
**Stratified**	**OCCIPITAL_WM**	**Occipital white matter**	**25(OH)D**	**Males**	**(87)**	**+261^c^**	**(67)**	**2.1e-04**	**+0.44**	**0.020**	**Yes**	**+0.45**	**0.001**
	**PARIETAL_WM**	**Parietal white matter**	**25(OH)D**	**Males**	**(87)**	**+486^c^**	**(129)**	**3.1e-04**	**+0.44**	**0.020**	**Yes**	**+0.45**	**0.001**
	**OCCIPITAL_WM**	**Occipital white matter**	**25(OH)D**	**>50**	**(80)**	**+205**	**(54)**	**3.2e-04**	**+0.37**	**0.020**	**Yes**	**+0.27**	**0.005**
	**PARIETAL_WM**	**Parietal white matter**	**25(OH)D**	**>50**	**(80)**	**+393^c^**	**(108)**	**5.4e-04**	**+0.37**	**0.020**	**Yes**	**+0.32**	**0.004**
	OCCIPITAL_WM	Occipital white matter	25(OH)D	AP	(132)	+156	(48)	1.3e-03	+0.25	0.050^d^	No	+0.26	0.004
	OCCIPITAL_WM	Occipital white matter	25(OH)D	Whites	(109)	+155	(49)	2.2e-03	+0.25	0.063^d^	No	+0.28	0.002
	FRONTAL_WM	Frontal white matter	25(OH)D	Males	(87)	+826**^c^**	(262)	2.3e-03	+0.38	0.063^d^	No	+0.42	0.003
	TEMPORAL_WM	Temporal white matter	25(OH)D	>50	(80)	+326**^c^**	(108)	3.5e-03	+0.29	0.084^d^	No	+0.23	0.024
	TEMPORAL_GM	Temporal gray matter	FOL	Whites	(109)	−354**^c^**	(123)	4.7e-03	−0.20	0.10	No	−0.26	0.004
	FRONTAL_GM	Fontal gray matter	B-12	AP	(132)	+13.4	(5.1)	9.7e-03	+0.17	0.18	No	+0.09	0.22

*25(OH)D, 25-hydroxyvitamin D; AP, Above poverty; B-12, serum cobalamin (vitamin B-12); FDR, False Discovery Rate; FOL, serum folate; FWER, FamilyWise Error Rate; GM, Gray Matter; SA, Sensitivity Analysis; SE, Standard Error; WM, White Matter*.

a *Values are adjusted linear regression coefficients β with associated SE, standardized beta, uncorrected p-values, corrected q-values (false discovery rate) and results of sensitivity analysis. (N) is the sample size in each analysis. Bolded rows correspond to statistically significant associations after correction for multiple testing, FWER < 0.05*.

b *Based on a sensitivity analysis further adjusting for selected socio-demographic, lifestyle and health-related factors after screening using machine learning techniques (see Supplemental Methods 2). Note that for visit 1 25(OH)D, no additional covariates were selected. For Folate and B-12 a reduced set of additional covariates were included and are listed in Supplemental Methods 2*.

c *P < 0.10 for null hypothesis that exposure × stratifying variable 2-way interaction term is =0 in the unstratified model with exposure and socio-demographic factors included as main effects*.

d *Finding considered a trend for passing FDR q-value correction at type I error of 0.10 per vitamin, model and stratification status while failing the FWER criterion, due to a standardized effect size (in absolute value) ≥0.20*.

For smaller ROI volumetric analysis (Table 3), 25(OH)D was significantly linked to larger left occipital pole volumes (FWER < 0.05, *b* = +0.35), overall and among individuals living above poverty, with a trend among men and Whites. Other stratum-specific trends were noted between 25(OH)D and right post-central gyrus volume in men, and parietal and occipital WM volume in men and the older group. Folate's relation with right temporal pole was detected among Whites (*p* < 0.05, *q* < 0.10 per vitamin, *b* = −0.34).

**Table 3:** Top 10 adjusted associations from model C, small sMRI regions vs. visit 1 exposures: serum 25(OH)D, folate and cobalamin (overall and stratified analysis) with uncorrected *P* < 0.05: ordinary least square brain scan-wide analyses on HANDLS 2004–2009 and HANDLS-SCAN 2011–2015^a^.

**Outcome (v_**scan**_)**	**Outcome description**	**Exposure (v_**1**_)**	**Stratum**	**(*N*)**	**β**	**(*SE*)**	***P***	**Standardized Beta (b)**	***q*-value**	**Passes FW Bonferroni correction**	**Standardized Beta (b): SA^b^**	***P*_**uncorr**_: SA**
**OVERALL**												
**Left_OCP_occipital_pole**	**Left occipital pole**	**25(OH)D**	**__**	**(186)**	**+15.70**	**(3.83)**	**6.3e-05**	**+0.31**	**0.026**	**Yes**	**+0.27**	**< 0.001**
occipital_lobe_WM_left	Occipital lobe, white matter, left	25(OH)D	__	(186)	+76.8	(20.7)	2.9e-04	+0.26	0.061^d^	No	+0.26	< 0.001
Right_PoG_post-central_gyrus	Post-central gyrus, right	25(OH)D	__	(186)	+34.8	(9.7)	4.3e-04	+0.27	0.061^d^	No	+0.27	0.001
parietal_lobe_WM_right	Parietal lobe, white matter, right	25(OH)D	__	(186)	+127.9	(38.1)	9.8e-04	+0.24	0.10^d^	No	+0.23	0.002
Left_PoG_post-central_gyrus	Post-central gyrus, left	25(OH)D	__	(186)	+34.1	(10.4)	1.3e-03	+0.25	0.11^d^	No	+0.25	0.002
Right_TrIFG_triangular_part_of_t	Triangular part of the inferior frontal gyrus, right	B-12	__	(240)	+0.45	(0.14)	2.2e-03	+0.20	0.13	No	+0.19	0.017
parietal_lobe_WM_left	Parietal lobe, white matter, left	25(OH)D	__	(186)	+123.4	(40.4)	3.1e-03	+0.22	0.13^d^	No	+0.21	0.007
occipital_lobe_WM_right	Occipital lobe, white matter, right	25(OH)D	__	(186)	+63.6	(21.1)	3.0e-03	+0.21	0.13^d^	No	+0.21	0.005
Right_TMP_temporal_pole	Right temporal pole	FOL	__	(240)	−35.5	(11.9)	2.7e-03	−0.19	0.13	No	−0.22	0.010
Anterior insula, right	Right_AIns_anterior_insula	B-12	__	(240)	+0.36	(0.12)	3.2e-03	+0.17	0.13	No	+0.13	0.071
**STRATIFIED**												
**Left_OCP_occipital_pole**	**Left occipital pole**	**25(OH)D**	**AP**	**(132)**	**+19.0^c^**	**(4.3)**	**2.0e-05**	**+0.35**	**0.07**	**Yes**	**+0.32**	**< 0.001**
Right_TMP_temporal_pole	Right temporal pole	FOL	Whites	(141)	−63.9^c^	(15.2)	4.8e-05	−0.34	0.08^d^	No	−0.42	< 0.001
Left_OCP_occipital_pole	Left occipital pole	25(OH)D	Men	(87)	+24.0	(5.8)	8.0e-05	+0.45	0.09^d^	No	+0.46	< 0.001
Left_OCP_occipital_pole	Left occipital pole	25(OH)D	Whites	(109)	+17.7	(4.5)	1.6e-04	+0.33	0.11^d^	No	+0.31	0.001
Right_PoG_post-central_gyrus	Right post-central gyrus	25(OH)D	Men	(87)	+64.4^c^	(16.6)	1.6e-04	+0.43	0.13^d^	No	+0.47	0.001
Parietal_lobe_WM_right	Right parietal lobe, White matter	25(OH)D	Men	(87)	+242.6^c^	(63.4)	2.6e-04	+0.45	0.13^d^	No	+0.46	0.001
occipital_lobe_WM_left	Occipital lobe, white matter, left	25(OH)D	>50	(80)	+107.7	(28.0)	3.4e-04	+0.37	0.14^d^	No	+0.28	0.004
parietal_lobe_WM_left	Parietal lobe, white matter, left	25(OH)D	>50	(80)	+201.8^c^	(53.6)	3.4e-04	+0.39	0.14^d^	No	+0.34	0.003
occipital_lobe_WM_right	Occipital lobe, white matter, right	25(OH)D	Men	(87)	+132.3^c^	(35.9)	4.1e-04	+0.43	0.14^d^	No	+0.44	0.001
Right_PHG_parahippocampal_gyrus	Right parahippocampal gyrus	FOL	Whites	(141)	−20.6^c^	(5.7)	4.2e-04	−0.27	0.14	No	−0.38	< 0.001

*25(OH)D, 25-hydroxyvitamin D; AP, Above poverty; B-12, serum cobalamin (vitamin B-12); FDR, False Discovery Rate; FOL, serum folate; FWER, FamilyWise Error Rate; GM, Gray Matter; SA, Sensitivity Analysis; SE, Standard Error; WM, White Matter*.

a *Values are adjusted linear regression coefficients β with associated SE, standardized beta, uncorrected p-values, corrected q-values (false discovery rate) and results of sensitivity analysis. (N) is the sample size in each analysis. Bolded rows correspond to statistically significant associations after correction for multiple testing, FWER < 0.05*.

b *Based on a sensitivity analysis further adjusting for selected socio-demographic, lifestyle and health-related factors after screening using machine learning techniques (see Supplemental Methods 2). Note that for visit 1 25(OH)D, no additional covariates were selected. For Folate and B-12 a reduced set of additional covariates were included and are listed in Supplemental Methods 2*.

c *P < 0.10 for null hypothesis that exposure × stratifying variable 2-way interaction term is =0 in the unstratified model with exposure and socio-demographic factors included as main effects*.

d *Finding considered a trend for passing FDR q-value correction at type I error of 0.10 per vitamin, model and stratification status while failing the FWER criterion, due to a standardized effect size (in absolute value) ≥0.20*.

In the dMRI analysis (Table 4 and Figure 2), both folate and 25(OH)D were significantly associated with better WMI, overall, in two key regions: Lower MD in the ALIC region for folate (*b* = −0.23, FWER < 0.05), and higher FA in the cingulum (cingulate gyrus) for 25(OH)D (FWER < 0.05, *b* = +0.31). No significant or trend associations were detected between vitamin B-12 and dMRI measures.

**Table 4:** Top 10 adjusted associations from model D, bilateral means of MD and FA from dMRI vs. visit 1 exposures: serum 25(OH)D, folate and cobalamin (overall and stratified analysis) with uncorrected *P* < 0.05: ordinary least square brain scan-wide analyses on HANDLS 2004–2009 and HANDLS-SCAN 2011–2015^a^.

**Outcome (v_**scan**_)**	**Outcome description**	**Exposure (v_**1**_)**	**Stratum**	**(*N*)**	**β**	**(*SE*)**	***P***	**Standardized Beta (b)**	***q*-value**	**Passes FW Bonferroni correction**	**Standardized Beta (b): SA^b^**	***P*_**uncorr**_: SA**
**OVERALL**												
**alic_b_tr**	**Anterior limb of the internal capsule, Mean diffusivity, bilateral mean**	**FOL**	**__**	**(240)**	**−5.64e-06**	**(1.56e-06)**	**3.8e-04**	**−0.23**	**0.074^d^**	**Yes**	**−0.26**	**0.003**
**cgc_b_fa**	**Cingulum (Cingulate Gyrus), fractional anisotropy, bilateral mean**	**25(OH)D**	**__**	**(185)**	**+0.0007**	**(0.0002)**	**4.1e-04**	**+0.31**	**0.074^d^**	**Yes**	**+0.28**	**0.002**
alic_b_fa	Anterior limb of the internal capsule, fractional anisotropy, bilateral mean	25(OH)D	__	(185)	+0.0006	(0.0002)	9.7e-04	+0.29	0.12^d^	No	+0.22	0.005
mcp_b_tr	Middle cerebellar peduncle, mean diffusivity, bilateral mean	B-12	__	(240)	−1.45e-07	(4.81e-08)	2.8e-03	−0.19	0.22	No	−0.18	0.019
mfowm_b_tr	Middle Fronto-Orbital WM, mean diffusivity, bilateral mean	FOL	**__**	(240)	−5.68e-06	(1.93e-06)	3.7e-03	−0.19	0.22	No	−0.23	0.019
cgh_b_fa	Cingulum (Hippocampus), fractional anisotropy, bilateral mean	25(OH)D	**__**	(185)	+0.0006	(0.0002)	3.9e-03	+0.25	0.22	No	+0.21	0.023
icp_b_fa	Inferior cerebellar peduncle, fractional anisotropy, bilateral mean	FOL	**__**	(240)	+0.0009	(0.0003)	4.5e-03	+0.19	0.22	No	+0.22	0.015
ss_b_fa	Sagittal Stratum, fraction anisotropy, bilateral mean	25(OH)D	**__**	(185)	+0.0004	(0.0002)	4.9e-03	+0.25	0.22	No	+0.20	0.010
mowm_b_tr	Middle Occipital WM, mean diffusivity, bilateral mean	FOL	__	(240)	−4.13e-06	(1.50e-06)	6.5e-03	−0.18	0.22	No	−0.17	0.024
put_b_tr	Putamen, mean diffusivity, bilateral mean	FOL	__	(240)	−4.22e-06	(1.54e-06)	6.5e-03	−0.18	0.22	No	−0.26	0.004
**STRATIFIED**												
alic_b_fa	Anterior limb of the internal capsule, fractional anisotropy, bilateral mean	25(OH)D	Whites	(109)	+0.0009 ^c^	(0.0002)	8.6e-05	+0.37	0.11^d^	No	+0.32	0.001
bcc_b_tr	Body of corpus callosum, Mean diffusivity, bilateral mean	25(OH)D	BP	(52)	−0.00002 ^c^	(4.43e-06)	8.7e-05	−0.53	0.11^d^	No	−0.61	0.001
cgc_b_fa	Cingulum (Cingulate Gyrus), fractional anisotropy, bilateral mean	25(OH)D	Whites	(109)	+0.0008	(0.0002)	1.1e-04	+0.39	0.11^d^	No	+0.36	< 0.001
sowm_b_fa	Superior Occipital WM, fractional anisotropy, bilateral mean	FOL	Males	(103)	+0.0016 ^c^	(0.0004)	2.1e-04	+0.39	0.12^d^	No	+0.31	0.007
unc_b_tr	Uncinate Fasciculus, mean diffusivity, bilateral mean	FOL	AA	(98)	2.2e-04^c^	(2.33e-06)	3.4e-04	−0.40	0.12^d^	No	−0.39	0.004
alic_b_tr	Anterior limb of the internal capsule, Mean diffusivity, bilateral mean	FOL	AP	(163)	−6.44e-06	1.72e-06	4.6e-04	−0.27	0.12^d^	No	−0.30	0.004
scc_b_tr	Splenium of Corpus Callosum, Mean diffusivity, bilateral mean	25(OH)D	BP	(52)	−0.000015 ^c^	(3.80e-06)	3.0e-04	−0.50	0.12^d^	No	−0.63	0.001
sowm_b_tr	Superior Occipital WM, mean diffusivity, bilateral mean	FOL	Males	(103)	−0.00001^c^	(3.53e-06)	4.6e-04	−0.37	0.15^d^	No	−0.38	< 0.001
alic_b_tr	Anterior limb of the internal capsule, Mean diffusivity, bilateral mean	FOL	>50 years	(96)	−0.00001^c^	(2.92e-06)	2.8e-04	−0.36	0.15^d^	No	−0.44	0.011
cgc_b_fa	Cingulum (Cingulate Gyrus), fractional anisotropy, bilateral mean	25(OH)D	BP	(52)	+0.00150	0.00040	5.8e-04	+0.57	0.17^d^	No	+0.59	0.003

*25(OH)D, 25-hydroxyvitamin D; AP, Above poverty; B-12, serum cobalamin (vitamin B-12); FOL, serum folate; FWER, FamilyWise Error Rate; GM, Gray Matter; SA, Sensitivity Analysis; SE, Standard Error; WM, White Matter*.

a *Values are adjusted linear regression coefficients β with associated SE, standardized beta, uncorrected p-values, corrected q-values (false discovery rate) and results of sensitivity analysis. (N) is the sample size in each analysis. Bolded rows correspond to statistically significant associations after correction for multiple testing, FWER < 0.05*.

b *Based on a sensitivity analysis further adjusting for selected socio-demographic, lifestyle and health-related factors after screening using machine learning techniques (see Supplemental Methods 2). Note that for visit 1 25(OH)D, no additional covariates were selected. For Folate and B-12 a reduced set of additional covariates were included and are listed in Supplemental Methods 2*.

c *P < 0.10 for null hypothesis that exposure × stratifying variable 2-way interaction term is =0 in the unstratified model with exposure and socio-demographic factors included as main effects*.

d *Finding considered a trend for passing FDR q-value correction at type I error of 0.10 per vitamin, model and stratification status while failing the FWER criterion, due to a standardized effect size (in absolute value) ≥0.20*.

Figure 2 highlights the strongest effect sizes and their associated uncorrected *p*-values observed in the dMRI analysis (Model D), through a series of volcano plots applied to the overall study sample, applied to v_1_ exposures. Effect sizes and direction were also visualized on standard ROI-specific brain images, for associations with *p*_uncorr_ < 0.05.

## Discussion

This study is among few that used a brain scan-wide analysis methodology to test associations of serum 25(OH)D, folate and cobalamin with brain volumes and WMI and the first to do so among socio-demographically diverse adults. The 3 vitamin status measures were systematically correlated with sMRI/dMRI brain markers, from low-to-high segmentation levels. We found statistically significant (FWER < 0.05) direct associations of 25(OH)D(v_1_) with total, occipital and parietal WM volumes, particularly among men and older participants and with left occipital pole volume, overall and among individuals living above poverty. Only trends were detected for cobalamin exposures (*q* < 0.10), while serum folate (v_1_) were associated with lower mean diffusivity (MD) in ALIC and with fractional anisotropy in the cingulum (cingulate gyrus), respectively, reflecting greater WMI, overall.

In terms of 25(OH)D and sMRI markers, vitamin D deficiency appears to be associated with smaller hippocampal subfields in MCI participants (Karakis et al., 2016; Al-Amin et al., 2019). Our study indicated that 25(OH)D was inversely linked to WM volumes, particularly in the left occipital pole. The occipital pole encompasses the primary visual cortex and contributes to language abilities (Charles et al., 1997; Melrose et al., 2009). Decline in verbal fluency has been related to lower 25(OH)D status (Beydoun et al., 2018; Goodwill et al., 2018). Relations of vitamin D deficiency with smaller WM volumes and poorer integrity were shown elsewhere (Buell et al., 2010; Prager et al., 2014; Annweiler et al., 2015b; Del Brutto et al., 2015). Vitamin D status was also associated with larger GM volumes (Brouwer-Brolsma et al., 2015), smaller ventricles (Annweiler et al., 2013) or not related to brain markers (Michos et al., 2014; Littlejohns et al., 2016). Our race-specific associations are notable, possibly due to genetic polymorphisms determining brain vitamin D status, which pending further studies, may be higher among Whites compared to AAs (Powe et al., 2013; Berg et al., 2015).

Among comparable ROI-specific dMRI studies, a cross-sectional study (Moon et al., 2015), found an inverse association between 25(OH)D and FA values near the inferior and superior longitudinal fasciculi, corpus callosum (genu), the anterior corona radiata, the ALIC and the cingulum bundle. Most regional FAs, particularly the ALIC and cingulum bundle (cingulate and hippocampus), were found to be positively associated with 25(OH)D in our study, with the cingulate gyrus exhibiting statistical significance.

Similarly, folate and cobalamin were previously linked to larger brain volumes (or slower atrophy), specifically within hippocampal and amygdala regions (Scott et al., 2004; Vogiatzoglou et al., 2008; Lee et al., 2016) and reduced WM lesion severity (De Lau et al., 2009; Pieters et al., 2009). In our study, cobalamin was related to occipital and temporal GM volumes, an association that was attenuated with full covariate-adjustment. B-6 and cobalamin intakes were also shown to spare GM atrophy, with specific association between cobalamin status and bi-lateral superior parietal sulcus (Erickson et al., 2008). Moreover, direct relationship between cobalamin status and regional GM volume (right precuneus, right post-central gyrus and left inferior parietal lobule) in AD was found mostly among ApoE4+ individuals (Lee et al., 2016). Our study showed a trend between increasing levels of cobalamin and larger parts of the inferior frontal gyrus [orbital (left); triangular (right)], known for its function in processing speech and language (Greenlee et al., 2007). A longitudinal study of adults found that lower cobalamin status, but not folate, was linked to increased rate of brain volume loss. A recent trial (VITACOG) conducted among MCI patients showed that GM regions vulnerable to AD, such as the medial temporal lobe, benefited from high-dose B vitamin supplementation by slowing atrophy rates over 2 years, though this pertained only to hyperhomocysteinemic individuals (Douaud et al., 2013), and this trial indicated that B vitamin supplementation can stabilize executive functions and reduce decline in global cognition, episodic and semantic memory (De Jager et al., 2012).

Novel are our findings that folate and 25(OH)D are related to greater white matter integrity, with folate being inversely related to MD in the ALIC region while 25(OH)D being related to higher FA in the cingulum (cingulate gyrus). While previous studies have linked vitamin D and folate deficiency to WM damage (Sachdev et al., 2002; Bleich and Kornhuber, 2003; Den Heijer et al., 2003; Dufouil et al., 2003; Scott et al., 2004; Censori et al., 2007; De Lau et al., 2009; Pieters et al., 2009; Buell et al., 2010; Prager et al., 2014; Annweiler et al., 2015b; Del Brutto et al., 2015; Moon et al., 2015; Wu et al., 2015; Lee et al., 2017), our study further specified most affected ROIs and target socio-demographic groups. The ALIC connects the thalamus with the frontal lobe, suggesting these nutrients can maintain cognitive functions that are reliant on frontothalamic connectivity, such as executive function (Schoenberg and Scott, 2011; Jacobs et al., 2013). Despite folate not being consistently associated with executive function or attention (Rosenberg, 2008), it was inversely related to depression (Bender et al., 2017) and reduced ALIC FA prevails in depressive disorders (Zou et al., 2008; Jia et al., 2010; Chen et al., 2016). Moreover, depressive symptoms increase dementia risk (Tan et al., 2019). Thus, future studies could explore mediation of the depression-AD relationship through ALIC FA and MD as the mechanism for folate supplementation prevention.

Our findings indicate that in certain sub-groups, folate may adversely affect volumetric markers, specifically the right temporal pole volume, thought to contribute to personal and episodic memories, also shown to be linked with empathy (Rankin et al., 2006). The literature shows an interaction between folate and cobalamin status, whereby high folate status coupled with cobalamin deficiency was associated with smaller GM volumes in the right middle occipital gyrus and the opercular part of the inferior frontal gyrus (Deng et al., 2017). Thus, abnormally high levels of folate may relate to poorer outcomes, though this finding may be spurious and due to chance, requiring replication in a larger meta-analytic studies.

Our study has several notable strengths. First, it examined the association between several AD-related nutritional biomarkers with brain structural sMRI and dMRI measures reflecting regional volumes and WMI, potentially underlying various neuropathologies. Moreover, while cross-sectional, this study provided 5–6 years of latency between exposure (nutritional biomarkers) and outcome (brain MRI measures) and secondarily tested stratum-specific heterogeneity and adjusting for multiple testing. Additionally, given that serum 25(OH)D was recently linked to lower intracranial volume (ICV) (Annweiler et al., 2015a), our detected positive association between 25(OH)D and brain volumes, including WM, may be conservative and under-estimated, and may be inflated upon ICV adjustment.

Nevertheless, study findings should be interpreted with caution given limitations. First, due to dMRI voxel size limitations, partial volume effects and possible contamination by nearby cerebral spinal fluid can occur, increasing FA and MD estimation errors. Second, timing of blood sample collection and measurement errors may have affected the sample distribution of serum 25(OH)D levels, with overestimation as a possibility as 10%-15% of the measured 25(OH)D values are in fact 24,25-dihydroxyvitamin D, which is recognized by the same antibody. Third, the latency between exposure and outcome could make the findings somewhat speculative when compared to a cohort study whereby baseline exposure is being tested against annualized change in outcome. The lack of a baseline sMRI/dMRI measure is a notable limitation of this study that should be remedied in further studies of comparable populations. Other potential limitations include the lack of other related serum measures, such as Hcy and vitamin B-6 in HANDLS, the lack of longer term markers, such as red blood cell folate, residual confounding particularly by physical activity which was not adequately measured at v_1_, non-participation selection bias, and a lower powered stratum-specific associations especially by race and poverty status. Due to differences in dietary intakes, absorption, utilization, distribution or other confounding conditions, circulating levels of target vitamins may not reflect their brain tissue levels, reducing their value as biomarkers. Moreover, our strongest findings implicate 25(OH)D as the main exposure, which may confound the association of serum folate with region-specific WMI. A larger meta-analytic study may be needed to disentangle those associations. Finally, external validity may be limited to inner US cities with similar racial/ethnic and socio-economic diversity as Baltimore City, as well as to middle-aged adults.

In summary, serum 25(OH)D status was consistently linked to larger occipital and parietal WM volumes and regional WMI. Pending longitudinal replication of our findings, future interventions should test vitamin D supplementation against regional volumetric and diffusion brain markers and mechanistic studies are needed to examine regional vulnerability to vitamin status.

## Data Availability Statement

Data are available upon request to researchers with valid proposals who agree to the confidentiality agreement as required by our Institutional Review Board. We publicize our policies on our website https://handls.nih.gov, which contains the code book for the parent study, HANDLS. Requests for data access may be sent to the PIs or the study manager, Jennifer Norbeck at norbeckje@mail.nih.gov. These data are owned by the National Institute on Aging at the National Institutes of Health. The Principal Investigators, have restricted public access to these data because (1) the study collects medical, psychological, cognitive, and psychosocial information on racial and poverty differences that could be misconstrued or willfully manipulated to promote racial discrimination; and (2) although the sample is fairly large, there are sufficient identifiers that the PIs cannot guarantee absolute confidentiality for every participant as we have stated in acquiring our confidentiality certificate. Analytic scripts and code book specific to HANDLS-SCAN can be obtained from the corresponding author upon request.

## Ethics Statement

The studies involving human participants were reviewed and approved by National Institute on Environmental Health Sciences IRB committee. The patients/participants provided their written informed consent to participate in this study.

## Author Contributions

MB contributed to the study concept, planned the analysis, conducted the data management and statistical analysis, conducted the literature review, wrote and revised the manuscript. DS planned the analysis, conducted the data management, conducted the literature review, wrote and revised the parts of the manuscript. SH conducted the literature search and review, assisted in statistical analysis, wrote the parts of the manuscript, and revised the manuscript. HB planned the analysis, conducted the literature review, wrote the parts of the manuscript, and revised the manuscript. LK, CD, RG, SS, and ME acquired the data, wrote and revised the parts of the manuscript. GE acquired the data, planned the analysis, assisted in data management and statistical analysis, wrote and revised the parts of the manuscript. AZ and SW acquired the data, the planned analysis, wrote and revised the parts of the manuscript.

## Conflict of Interest

The authors declare that the research was conducted in the absence of any commercial or financial relationships that could be construed as a potential conflict of interest.

## Footnotes

^1^Siemens Centaur. Available online at: https://www.siemens-healthineers.com/en-us/immunoassay/systems.

^2^Diagnostics, Q. *Vitamin B-12 (Cobalamin) and Folate Panel*. Available online at: https://testdirectory.questdiagnostics.com/test/test-detail/7065/vitamin-b12-cobalamin-and-folate-panel-serum?cc=MASTER (accessed October 21, 2019).

^3^NHANES 2003–2004. Available online at: https://www.cdc.gov/nchs/data/nhanes/nhanes_03_04/l06vid_c_met_Vitamin_D.pdf (accessed December 16, 2019).

^4^Diasorin. Available online at: https://diasoringroup.com/en?gclid=EAIaIQobChMI3JLjuq265gIVhIvICh3G-QcTEAAYASAAEgLz-fD_BwE (accessed December 16, 2019).

## Abbreviations

AA: African Americans
ALIC: Anterior Limb of the Internal Capsule
C-TRIM: Core for Translational Research in Imaging @ Maryland
DTI, Diffusion Tensor Imaging, dMRI: Diffusion MRI
FA: Fractional Anisotropy
FWER, Familywise Error Rate, FDR: False Discovery Rate
FLAIR: Fluid-Attenuated Inversion Recovery
FOV: Field of View
GM: Gray Matter
HANDLS: Healthy Aging in Neighborhoods of Diversity across the Life Span study
HS: High School
LRP2: Megalin gene
MP-RAGE: Magnetization prepared rapid gradient echo
MRI: Magnetic Resonance Imaging
MD: Mean Diffusivity
MRV: Medical Research Vehicle
MMSE: Mini-Mental State Examination
MICO: Multiplicative intrinsic component optimization
MUSE: Multi-atlas region Segmentation utilizing Ensembles
OCM: One-Carbon Metabolism
ROI: Regions of Interest
25(OH)D: Serum 25-hydroxyvitamin D
FOL: Serum folate
B-12: Serum vitamin B-12
Hcy: Homocysteine
SA: Sensitivity Analysis
sMRI: Structural MRI
TR: TRACE
US: United States
*VDR*: Vitamin D receptor gene
WMI: White Matter Integrity
WM: White Matter.
