# Corrigendum: Vitamin D, Folate, and Cobalamin Serum Concentrations Are Related to Brain Volume and White Matter Integrity in Urban Adults

**DOI:** 10.3389/fnagi.2021.660049

**Published:** 2021-04-19

**Authors:** May A. Beydoun, Danielle Shaked, Sharmin Hossain, Hind A. Beydoun, Leslie I. Katzel, Christos Davatzikos, Rao P. Gullapalli, Stephen L. Seliger, Guray Erus, Michele K. Evans, Alan B. Zonderman, Shari R. Waldstein

**Affiliations:** ^1^Laboratory of Epidemiology and Population Sciences, The National Institute on Aging (NIA) the Intramural Research Program (IRP), The National Institutes of Health (NIH), Baltimore, MD, United States; ^2^Department of Psychology, University of Maryland, Baltimore County, MD, United States; ^3^Department of Research Programs, Fort Belvoir Community Hospital, Fort Belvoir, VA, United States; ^4^Geriatric Research Education and Clinical Center, Baltimore VA Medical Center, Baltimore, MD, United States; ^5^Division of Gerontology & Geriatric Medicine, Department of Medicine, University of Maryland School of Medicine, Baltimore, MD, United States; ^6^Section for Biomedical Image Analysis, Department of Radiology, University of Pennsylvania, Philadelphia, PA, United States; ^7^Department of Diagnostic Radiology, University of Maryland School of Medicine, Baltimore, MD, United States; ^8^Division of Nephrology, Department of Medicine, University of Maryland School of Medicine, Baltimore, MD, United States

**Keywords:** 25-hydroxyvitamin D, folate, cobalamin, brain volumes, white matter integrity, cognitive aging, health disparities

In the original article, there were several errors related to the supplemental materials that are now omitted in this new version.

A coding error related to the mixed-effects regression models to estimate slopes has made part of the analysis on change in vitamin D, folate and B-12 irrelevant and redundant to the main results.

Supplemental Method 2 is no longer relevant. Supplemental Method 3 is now Supplemental Method 2.

Pages 12-13 Table in the supplement (now supplemental method 2): Rows for Vitamin D(slope), folate(slope) and B-12(slope) are no longer relevant and were omitted.

Supplement, Pages 28-36: Supplemental Tables 3-5 is now omitted and is no longer part of the main findings.

Additional changes were also made due to previous omission of an important finding in the text.

The specific text corrections are shown below.

Corrections have been made to the *Abstract, Methods, Results*, and *Conclusions* sub-sections. The corrected Abstract is shown below:

**Background and objectives**: Lower vitamin status has been linked to cognitive deficits, pending mechanistic elucidation. Serum 25-hydroxyvitamin D [25(OH)D], folate and cobalamin were explored against brain volumes and white matter integrity (WMI).

**Methods**: Two prospective waves from Healthy Aging in Neighborhoods of Diversity Across the Life Span (HANDLS) study were primarily used [Baltimore, City, MD, 2004–2015, *N* = 183–240 urban adults (Age_v1_: 30–64 years)]. Serum vitamin 25-hydroxyvitamin D [25(OH)D], folate and cobalamin concentrations were measured at visits 1 (v_1_: 2004–2009), while structural and diffusion Magnetic Resonance Imaging (sMRI/dMRI) outcomes were measured at vscan: 2011–2015. Top 10 ranked adjusted associations were corrected for multiple testing using familywise Bonferroni (FWER < 0.05) and false discovery rates (FDR, *q*-value < 0.10).

**Results**: We found statistically significant (FWER < 0.05; β±SE) direct associations of 25(OH)D(v_1_) with WM volumes [overall: +910 ± 336/males: +2,054 ± 599], occipital WM; [overall: +140 ± 40, males: +261 ± 67 and Age_v1_ > 50 years: +205 ± 54]; parietal WM; [overall: +251 ± 77, males: +486 ± 129 and Age_v1_ > 50 years: +393 ± 108] and left occipital pole volume [overall: +15.70 ± 3.83 and above poverty: 19.0 ± 4.3]. Only trends were detected for cobalamin exposures (*q* < 0.10), while serum folate (v_1_) was associated with lower mean diffusivity (MD) in the Anterior Limb of the Internal Capsule (ALIC), reflecting greater WMI, overall, while regional FA (e.g., cingulum gyrus) was associated with greater 25(OH)D concentration.

**Conclusions**: Among urban adults, serum 25(OH)D status was consistently linked to larger occipital and parietal WM volumes and greater region-specific WMI. Pending longitudinal replication of our findings, randomized controlled trials of vitamin D supplementation should be conducted against brain marker outcomes.

A correction has been made to *Methods and Materials, Database, Paragraph 4*. The corrected paragraph is shown below:

This study analyzed nutritional biomarker data from visit 1 (v_1_: 2004–2009) in relation to follow-up data measured in a sub-sample of N_max_ = 258 participants within the HANDLS SCAN sub-study (vscan: 2011–2015) (Waldstein et al., 2017). Exposure variables were measured during the Medical Research Vehicle (MRV) examinations; outcomes were MRI measures of brain volume and WMI at vscan (Waldstein et al., 2017). The mean follow-up time between visit 1 and vscan was 5.70 years ± 1.90.

A correction has also been made to *Methods and Materials, Vitamin Status Measures, Paragraph 3*. The corrected paragraph is shown below:

Dietary and supplemental intakes of vitamin D, folate and cobalamin were shown to moderately correlate with their corresponding serum biomarkers in HANDLS and national surveys (Beydoun et al., 2010a,b; Beydoun et al., 2018). Moreover, moderate-to-strong correlations were detected for all three biomarkers (Pearson's *r* > 0.30), notably v_1_ vs. v_2_ values for each vitamin in the HANDLS sample: 25(OH)D (*r* = 0.44, *n* = 1,462); folate (*r* = 0.44, *n* = 1,944); cobalamin (*r* = 0.55, *n* = 1,994). We also describe categorical exposures with cutoffs reflecting vitamin insufficiency or deficiency (Snow, 1999; Thacher and Clarke, 2011; World Health Organization, 2015).

A correction has also been made to *Methods and Materials, Covariates*. The corrected paragraph is shown below:

All models were adjusted for baseline examination age (y), sex (male = 1, female = 0), race (AA = 1, White = 0), self-reported household income either <125% or ≥125% of the 2004 Health and Human Services poverty guidelines (termed poverty status) (US Department of Health & Human Services, 2019), and time (days) between baseline MRV visit and MRI scan visit. Models were independently stratified by age ( ≤ 50 vs. >50 years), sex, race, or poverty status. Additional covariates were entered in a sensitivity analysis when independently associated with each exposure of interest (see Supplemental Method 2).

Corrections have been made to *Methods and Materials, Statistical Analysis, Paragraph 2*. The corrected paragraph is shown below:

For uncorrected *p*-values, Type I error <0.05 was used for significance. To adjust for multiple testing two methods were used: (1) Familywise Bonferroni (error rate) correction (FWER) which adjusted for multiplicity in brain MRI measures, assuming each set of modeling approach (Models A-D and stratification status) applied to each serum vitamin [25(OH)D, folate and cobalamin] to be separate hypotheses, (2) false discovery rate (*q*-value) which only considered the four approaches/stratification status as separate hypotheses (i.e., Models A-D, and stratification status), thus combining the 3 vitamin exposures upon correction. Moreover, the top 10 adjusted associations from each analysis were presented if *p*_uncorr_ < 0.05, showing the main parameter estimate and its standard error (SE), the uncorrected *p*-values, the FDR *q*-values and FWER status (Yes = passed correction, No = did not pass) and the standardized effect size *b*. Top 10 associations were considered statistically significant if passing FWER correction for a specific vitamin, model and stratification status (yes vs. no) at type I error of 0.05. Results with FDR *q*-value < 0.10 per model and stratification status while failing the FWER criterion were considered a trend. Additionally, when passing FDR *q*-value correction at type I error of 0.10 per vitamin, model and stratification status while failing the FWER criterion, an effect was considered a trend if |*b*| ≥0.20. Among selected stratified models (top 10 findings), formal effect modification testing was conducted by including 2-way interaction terms between exposure and each socio-demographic factor in the non-stratified model. A Type I error of 0.10 was used for 2-way interaction terms due to reduced statistical power (Selvin, 2004). In addition, the main analyses with v_1_ exposures and minimal socio-demographic adjustment, sensitivity analyses were conducted with additional adjustments (Supplemental Method 2).

Corrections have been made to *Results, Paragraph 2*. The corrected paragraph is shown below:

Top 10 adjusted associations with uncorrected *p* < 0.05 from ordinary least square brain scan-wide analyses are presented in **Tables 2–4** and **Figure 2**. Among significant findings (FWER < 0.05) in the main analysis (**Table 2**), serum 25(OH)D was directly associated with larger WM volumes [overall (β = +910 ± 336, *p* = 0.007, *q* = 0.067, passed FW Bonferroni correction), effect size *b* = 0.19], with a stronger effect size among men (*b* = 0.41). This association was specific to occipital and parietal WM, with a moderate effect size (*b* = +0.23–0.25, *q* < 0.05, passed FW Bonferroni correction) in the overall sample, men and the older group. A trend toward a direct association was also detected between 25(OH)D and total brain volume in the overall sample, in men and those in the older group. Among trends (*q*-value < 0.10), temporal and occipital WM volumes were directly associated with 25(OH)D, in Whites and individuals living above poverty, respectively. Most of these 25(OH)D vs. larger ROIs associations were not altered when additional covariates were entered in a sensitivity analysis (**Table 2**). Higher cobalamin exhibited a trend association with larger total brain, total GM, frontal and occipital GM volumes in the overall sample (*q*-value < 0.10), becoming null after adjustment for 25(OH)D and other covariates (see Supplemental Method 2).

Corrections have also been made to *Results, Paragraphs 3* and *4*. The corrected paragraphs are shown below:

For smaller ROI volumetric analysis (**Table 3**), 25(OH)D was significantly linked to larger left occipital pole volumes (FWER < 0.05, *b* = +0.35), overall and among individuals living above poverty, with a trend among men and Whites. Other stratum-specific trends were noted between 25(OH)D and right post-central gyrus volume in men, and parietal and occipital WM volume in men and the older group. Folate's relation with right temporal pole was detected among Whites (*p* < 0.05, *q* < 0.10 per vitamin, *b* = −0.34).

In the dMRI analysis (**Table 4** and **Figure 2**), both folate and 25(OH)D were significantly associated with better WMI, overall, in two key regions: Lower MD in the ALIC region for folate (*b* = −0.23, FWER < 0.05), and higher FA in the cingulum (cingulate gyrus) for 25(OH)D (FWER < 0.05, *b* = +0.31). No significant or trend associations were detected between vitamin B-12 and dMRI measures.

Corrections have been made to *Discussion, Paragraph 1*. The corrected paragraph is shown below:

This study is among few that used a brain scan-wide analysis methodology to test associations of serum 25(OH)D, folate and cobalamin with brain volumes and WMI and the first to do so among socio-demographically diverse adults. The 3 vitamin status measures were systematically correlated with sMRI/dMRI brain markers, from low-to-high segmentation levels. We found statistically significant (FWER < 0.05) direct associations of 25(OH)D(v_1_) with total, occipital and parietal WM volumes, particularly among men and older participants and with left occipital pole volume, overall and among individuals living above poverty. Only trends were detected for cobalamin exposures (*q* < 0.10), while serum folate (v_1_) were associated with lower mean diffusivity (MD) in ALIC and with fractional anisotropy in the cingulum (cingulate gyrus), respectively, reflecting greater WMI, overall.

Corrections have also been made to *Discussion, Paragraph 3*. The corrected paragraph is shown below:

Among comparable ROI-specific dMRI studies, a cross-sectional study (Moon et al., 2015), found an inverse association between 25(OH)D and FA values near the inferior and superior longitudinal fasciculi, corpus callosum (genu), the anterior corona radiata, the ALIC and the cingulum bundle. Most regional FAs, particularly the ALIC and cingulum bundle (cingulate and hippocampus), were found to be positively associated with 25(OH)D in our study, with the cingulate gyrus exhibiting statistical significance.

Corrections have also been made to *Discussion, Paragraph 5*. The corrected paragraph is shown below:

Novel are our findings that folate and 25(OH)D are related to greater white matter integrity, with folate being inversely related to MD in the ALIC region while 25(OH)D being related to higher FA in the cingulum (cingulate gyrus). While previous studies have linked vitamin D and folate deficiency to WM damage (Sachdev et al., 2002; Bleich and Kornhuber, 2003; Den Heijer et al., 2003; Dufouil et al., 2003; Scott et al., 2004; Censori et al., 2007; De Lau et al., 2009; Pieters et al., 2009; Buell et al., 2010; Prager et al., 2014; Annweiler et al., 2015b; Del Brutto et al., 2015; Moon et al., 2015; Wu et al., 2015; Lee et al., 2017), our study further specified most affected ROIs and target socio-demographic groups. The ALIC connects the thalamus with the frontal lobe, suggesting these nutrients can maintain cognitive functions that are reliant on frontothalamic connectivity, such as executive function (Schoenberg and Scott, 2011; Jacobs et al., 2013). Despite folate not being consistently associated with executive function or attention (Rosenberg, 2008), it was inversely related to depression (Bender et al., 2017) and reduced ALIC FA prevails in depressive disorders (Zou et al., 2008; Jia et al., 2010; Chen et al., 2016). Moreover, depressive symptoms increase dementia risk (Tan et al., 2019). Thus, future studies could explore mediation of the depression-AD relationship through ALIC FA and MD as the mechanism for folate supplementation prevention.

Corrections have also been made to *Discussion, Paragraphs 7, 8*, and *9*. The corrected paragraphs are shown below:

Our study has several notable strengths. First, it examined the association between several AD-related nutritional biomarkers with brain structural sMRI and dMRI measures reflecting regional volumes and WMI, potentially underlying various neuropathologies. Moreover, while cross-sectional, this study provided 5–6 years of latency between exposure (nutritional biomarkers) and outcome (brain MRI measures) and secondarily tested stratum-specific heterogeneity and adjusting for multiple testing. Additionally, given that serum 25(OH)D was recently linked to lower intracranial volume (ICV) (Annweiler et al., 2015a), our detected positive association between 25(OH)D and brain volumes, including WM, may be conservative and under-estimated, and may be inflated upon ICV adjustment.

Nevertheless, study findings should be interpreted with caution given limitations. First, due to dMRI voxel size limitations, partial volume effects and possible contamination by nearby cerebral spinal fluid can occur, increasing FA and MD estimation errors. Second, timing of blood sample collection and measurement errors may have affected the sample distribution of serum 25(OH)D levels, with overestimation as a possibility as 10%-15% of the measured 25(OH)D values are in fact 24,25-dihydroxyvitamin D, which is recognized by the same antibody. Third, the latency between exposure and outcome could make the findings somewhat speculative when compared to a cohort study whereby baseline exposure is being tested against annualized change in outcome. The lack of a baseline sMRI/dMRI measure is a notable limitation of this study that should be remedied in further studies of comparable populations. Other potential limitations include the lack of other related serum measures, such as Hcy and vitamin B-6 in HANDLS, the lack of longer term markers, such as red blood cell folate, residual confounding particularly by physical activity which was not adequately measured at v_1_, non-participation selection bias, and a lower powered stratum-specific associations especially by race and poverty status. Due to differences in dietary intakes, absorption, utilization, distribution or other confounding conditions, circulating levels of target vitamins may not reflect their brain tissue levels, reducing their value as biomarkers. Moreover, our strongest findings implicate 25(OH)D as the main exposure, which may confound the association of serum folate with region-specific WMI. A larger meta-analytic study may be needed to disentangle those associations. Finally, external validity may be limited to inner US cities with similar racial/ethnic and socio-economic diversity as Baltimore City, as well as to middle-aged adults.

In summary, serum 25(OH)D status was consistently linked to larger occipital and parietal WM volumes and regional WMI. Pending longitudinal replication of our findings, future interventions should test vitamin D supplementation against regional volumetric and diffusion brain markers and mechanistic studies are needed to examine regional vulnerability to vitamin status.

The authors apologize for these errors and state that they do not change the scientific conclusions of the article in any way. The original article and Supplementary Material have been updated.
