# Supplementary material for: Vitamin D, Folate, and Cobalamin Serum Concentrations Are Related to Brain Volume and White Matter Integrity in Urban Adults
Source: Front Aging Neurosci. 2020 May 25;12:140. doi: 10.3389/fnagi.2020.00140 (PMC7261885; doi:10.3389/fnagi.2020.00140)
Supplement: Supplementary file 1 [file Data_Sheet_1.docx]

**Vitamin D, folate and cobalamin status and change are related to brain volume and white matter integrity in urban adults**

Beydoun et. al.

**ONLINE SUPPLEMENTARY MATERIAL**

**Supplemental Method 1. Brain structural/diffusion (s) magnetic resonance imaging (MRI) and diffusion (d) MRI detailed description:**

**HANDLS description**

**sMRI**

The T1-weighted MP-RAGE images covered the whole brain in a sagittal plane at a thickness of 1.2 mm for 160 slices (TR/TE/TI=2300/2.9/900 ms; FOV 25.6cm). These images were converted from sagittal to axial sections for comparative purposes.

The Section for Biomedical Image Analysis at the University of Pennsylvania developed in-house techniques to preprocess structural MRI scans. First, extra-cranial material on the T1-weighted images was removed using a multi-atlas registration method requiring minimal correction by hand (Doshi et al., 2013). Multiplicative intrinsic component optimization (MICO) method was used to correct for bias (Li et al., 2014). MUlti-atlas region Segmentation utilizing Ensembles (MUSE), segmented pre-processed images into a set of anatomical regions of interest (ROIs) (Doshi et al., 2016). MUSE integrates a broad ensemble of labeled templates by using a number of warping algorithms, regularization atlases and parameters (Doshi et al., 2016).

**dMRI**

Isotropic resolution images were acquired with an in-plane resolution of 2x2 mm and 2 mm slice thickness over a 22.4 cm FOV. A total of 66 slices at a TE = 122ms, TR = 3300ms, and flip angle = 90o were used. Eddy current effects were reduced by using bipolar diffusion. Diffusion weighting scheme was a 2-shell (b = 1000, 2500), optimized for uniform sampling of each shell and non-overlapping diffusion directions of 60 and 120 for each shell, respectively, and 6 b0 volumes. The image acquisition time was ten minutes.

Joint Linear Minimum Mean Squared Error denoising software (jLMMSE; Tristan-Vega and Aja-Fernandez, 2010) was used to de-noise the raw DWI data. The DT images were reconstructed by fitting the de-noised DWI data using multivariate linear fitting. Motion correction was conducted with FSL’ s “eddycorrect” tool (Andersson and Sotiropoulos, 2016).

Fractional Anisotropy (FA) – a widely established method for quantifying WMI sensitive to the degree of myelination, density, and organization of WM – was used to determine directionality of water diffusion in the brain. It measures the degree of anisotropy of the diffusion at the voxel level. It is derived from the variance of the average of the three eigenvalues of the diffusion tensor that are used to compute FA values, ranging from 0 to 1; 0 indicates completely unrestricted diffusion, and 1 denotes completely restricted diffusion. Computing the sum of the eigenvalues of the diffusion tensor yields the TR or mean diffusivity (MD), with a higher value indicative of poorer WMI. (Jones, 2008).

**Quality assurance**

The Core for Translational Research in Imaging @ Maryland (C-TRIM), managed by the Department of Diagnostic Radiology at UMB’s School of Medicine, has instituted several quality control measures to ensure highest level of quality (and safety). The research dedicated scanner undergoes routine American College of Radiology mandated daily quality assurance(Mulkern et al., 2008). In addition, the AD Neuroimaging Initiative phantom is used to assess weekly signal-to-noise ratio and monthly structural distortions(Gunter et al., 2009). We periodically check the reliability of diffusion data by using the National Institutes of Standards and Technology diffusion phantom to ensure that the diffusion measurements from diffusion MRI are stable(phantom)

**Supplemental Method 2: Additional covariates, LASSO regression and multiple imputations**

**A. Additional covariates:**

**A.1. Socio-demographic**

Additional socio-demographic confounders included educational attainment (0≤High School (HS); 1=HS and 2≥HS), the Wide Range Achievement Test (WRAT) letter and word reading subtotal scores to measure literacy and marital status (1=married, 0=not married) (Beydoun et al., 2018).

**A.2. Lifestyle**

**Smoking and drug use**

Current use of opiate, marijuana or cocaine use (“current” *vs*. “never or former”) and smoking status (“current” vs. “never or former”) were considered.

**Adiposity measures**

Measured body mass index (BMI, kg/m^2^), waist circumference and waist-hip-ratio were considered among potential confounders.

**Healthy Eating Index 2010-**

The Healthy Eating Index (HEI-2010) total score, based on two 24-hr recalls administered at baseline, was used as a measure of overall dietary quality. See steps for calculating HEI-2010 at <http://appliedresearch.cancer.gov/tools/hei/tools.html> and <http://handls.nih.gov/06Coll-dataDoc.html>.

**Dietary Approaches to Stop Hypertension (DASH)**

The score for DASH diet adherence, based on 8 nutrients, was determined for each participant using the formula reported by Mellen *et al*.(Mellen et al.). The nine target nutrients were total fat, saturated fat, protein, fiber, cholesterol, sodium, calcium, magnesium, and potassium. Micronutrient goals were expressed per 1000 kcal. The total DASH score was generated by the sum of all nutrient targets met. If the participant achieved the DASH target for a nutrient, a value of 1 was assigned, and if the intermediate target for a nutrient was achieved, a value of 0.5 was assigned. A value of zero was assigned if neither target was met. The maximum DASH score was 9; individuals meeting approximately half of the DASH targets (DASH score = 4.5) were considered DASH adherent (Mellen et al.).

**Mean Adequacy Ratio (MAR)**

Diet quality was also assessed using Nutrient Adequacy Ratio (NAR) and Mean Adequacy Ratio (MAR) scores(Murphy et al., 2006;Fanelli Kuczmarski et al., 2013). The NAR score was determined by taking each participant’s daily intake of a nutrient divided by the Recommended Dietary Allowance (RDA) for that nutrient. NAR scores were determined for 17 micronutrients: vitamins A, C, D, E, B_6_, B_12_, folate, iron, thiamin, riboflavin, niacin, copper, zinc, calcium, magnesium, phosphorus, and selenium. The RDA was adjusted for participants’ ages and sexes and vitamin C was adjusted for smokers(Murakami et al., 2019). The NAR score was converted into a percent with values exceeding 100 truncated to 100. MAR scores were calculated by averaging the NAR scores: MAR= (∑NAR scores)/17(Fanelli Kuczmarski et al., 2018).
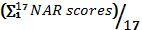
 NAR and MAR were calculated separately for each daily-intake and then averaged. MAR scores, based on food intakes only, were used as the nutrient-based diet quality variable.

**Supplemental use**

The HANDLS dietary supplement questionnaire was adapted from the 2007 NHANES instrument.(Centers for Disease Control and Prevention, 2007) HANDLS participants provided supplement bottles during their dietary interview at the follow-up visit only (i.e. visit 2). Information on Over-The-Counter (OTC) vitamin and mineral supplements, antacids, prescription supplements, and botanicals were reported, and supplement users were asked about dose strength, dose amount consumed, length of supplement use (converted to days), frequency of use (daily, monthly, seasonally, annually), and if each supplement was taken the day prior to interview(Beydoun et al., 2018).

A HANDLS dietary supplement database was developed by trained nutritionists and registered dietitians. This database consisted of 4 files integrated to generate daily intake of each nutrient consumed by a dietary supplement user. [See detailed description at the HANDLS study website: https://handls.nih.gov/].

**Depressive symptoms**

Depressive symptoms were operationalized using the CES-D, at both baseline and follow-up. The 20-item CES-D is a self-reported symptom rating scale assessing affective and depressed mood.(Radloff, 1977) A score of ≥16 on the CES-D is reflective of elevated depressive symptoms (EDS), (Ramos et al., 2004) and predicts clinical depression based on the Diagnostic and Statistical Manual, fourth edition (DSM-IV) criteria.(Myers and Weissman, 1980) Four CES-D sub-domains exhibiting an invariant factor structure between The National Health and Nutrition Examination Survey I and pilot HANDLS data (Nguyen et al., 2004) were computed. We tested our hypotheses using total and domain-specific CES-D scores: **(1)** Somatic complaints; **(2)** Depressive affect; **(3)** Positive affect and **(4)** Interpersonal problems.(Nguyen et al., 2004)

**A.3. Health-related**

Baseline chronic conditions included self-reported history and biomarker-based measurement (as well as medication-based) of type 2 diabetes, hypertension, dyslipidemia, cardiovascular disease and inflammatory disease. Dyslipidemia was based on a combination of self-report, HDL, total cholesterol, triglyceride criteria as well as statin use. Similarly, type 2 diabetes was determined using a combination of self-report, serum glucose criteria and medication, as was the case for hypertension. In addition, a composite of cardiovascular disease history was added in which self-reported stroke, congestive heart failure, non-fatal myocardial infarction or atrial fibrillation were considered and combined into a yes/no variable. Similarly, inflammatory disease was a binary composite of multiple sclerosis, systemic lupus, gout, rheumatoid arthritis, psoriasis, Thyroid disorder and Crohn’s disease. The use of NSAIDs (NSAIDs, prescription and over-the-counter) over the past two weeks as well as use of statins were considered separately as potential covariates.

**A.4. Other biomarkers**

All laboratory tests selected for this study were done at Quest Diagnostics, Chantilly, VA.

**Serum cholesterol and atherogenic indices**

Total cholesterol (TC), High density lipoprotein-cholesterol (HDL-C) and Triacylglycerols (TA) were assessed using a spectrophotometer (Olympus 5400). Low density lipoprotein-cholesterol (LDL-C) was calculated as TC-(HDL-C+TA/5) and directly measured in a sub-sample (N=236) also using a spectrophotometer (Olympus 5400). The correlation between those with baseline calculated LDL-C and those with measured LDL-C was r~0.95. From these measures, two relative measures were obtained, namely TC:HDL-C and LDL-C:HDL-C ratios. Those two relative measures, also termed “atherogenic indices” were previously studied in relation to various cardiovascular outcomes and were found to be positively associated with measures of atherosclerosis and coronary heart disease. (Nair et al., 2009;Manickam et al., 2011;Hisamatsu et al., 2014)

**Serum uric acid (SUA)**

SUA measurements are useful in the diagnosis and treatment of renal and metabolic disorders, including renal failure, gout, leukemia, psoriasis, starvation or other wasting conditions, and in patients receiving cytotoxic drugs. Using 1 ml of fasting blood serum, uric acid was measured using a standard spectrophotometry method. The reference range for adult men is 4.0-8.0 mg/dL, whereas for women, this range is cited as 2.5-7.0 mg/dL. (<http://www.questdiagnostics.com/testcenter/TestDetail.action?ntc=905>) Other reference ranges were also recently suggested and depend on the menopausal status of women. Those reference ranges are based on predictive value for gout outcomes among healthy individuals and do not necessarily predict other pathologies. Thus, based on recent research evidence, a “normal” SUA value is suggested to be <6.0 mg/dL for all healthy adult individuals.

**Serum albumin**

Using 0.5-1 mL sample of plasma prepared with heparin and refrigerated for up to 30 days, albumin was measured with spectrophotometry, with an expected reference range of 3.6-5.1 g/dL(Beydoun et al., 2016b;Beydoun et al., 2019).

**High sensitivity C-reactive protein (CRP)**

High sensitivity CRP (hs-CRP) was analyzed with an immunoturbidimeter (Siemens/Behring Nephelometer II), using 0.5-1 mL of plasma, with the range 1-10 mg/dL indicating average or high cardiovascular risk and >10 mg/dL suggestive of an infection or a chronic inflammation.

**Serum creatinine**

Using participant fasting venous blood specimens, baseline serum creatinine was measured at the National Institute on Aging, Clinical Research Branch Core Laboratory, using a modified kinetic Jaffe method (CREA method, Dade Dimension X-Pand Clinical Chemistry System, Siemens Healthcare Diagnostics Inc., Newark, DE) for a small group of participants (n=88); while the majority of participants (n=1,528) had baseline serum creatinine analyzed at Quest Diagnostics, Inc. by isotope dilution mass spectrometry (IDMS) (Olympus America Inc., Melville, NY) and standardized to the reference laboratory, Cleveland Clinic. While inter-assay coefficients of variation (CV) for this sample could not be calculated due to the use of only one or the other measurement of creatinine at baseline, only intra-assay CVs (mean/SD) could be estimated and those were 0.192 and 0.187 for the CREA and the IDMS methods, respectively.

**HbA1c**

Glycated hemoglobin is derived from the nonenzymatic addition of glucose to amino groups of hemoglobin. HbA1c is a specific glycated hemoglobin that results from the attachment of glucose to the N-terminal valine of the hemoglobin b-chain. Numerous assays were subsequently developed to measure glycated hemoglobins. The principle of all methods is to separate the glycated and nonglycated forms of hemoglobin(Beydoun et al., 2016a). This can be accomplished based on differences in charge (usually by HPLC) or structure (usually immunoassays

or boronate affinity chromatography). In this study, the method adopted was HPLC (Quest diagnostics).

**White blood cell inflammatory markers**

Fasting blood samples were collected from participants at baseline and follow-up to determine total white blood cell count, (K/mm^3^), using electronic Cell Sizing, counting, cytometry and microscopy. (<http://www.questdiagnostics.com/testcenter/TestDetail.action?ntc=7064>).

**Red cell distribution width (RDW), hemoglobin and other iron status measures**

*RDW*

RDW was measured by automated Coulter DXH 800 hematology analyzer as part of peripheral complete blood count (Beckman Coulter, Brea, CA), and was expressed as coefficient of variation (%) of red blood cell volume distribution. Regular calibration was performed every 3 months on the hematology analyzer and quality control was performed according to the manufacturer’s recommendations.(Diagnostics) There are usually two RDW measurements used for clinical purposes, namely the RDW-coefficient of variation (CV, unit: %), which we used in this study, and the RDW-Standard Deviation (SD, unit: fL) from which RDW-CV is derived. In fact, RDW-CV=RDW-SD×100/MCV, where MCV is the mean cell volume. The normal range for RDW-CV is 11.0 - 15.0%. Thus, the RDW-CV (%) depends on both the width of the distribution (normal range: 40-55 fL) curve and the MCV.(techs, 2019)

*Hemoglobin (Hb)*

Similarly, using electronic cell sizing/cytometry/microscopy, Hb was assayed from a sample of 1 ml of blood drawn from participants after overnight fast, and refrigerated up to 6 days (Quest diagnostics).

*Other iron status markers*

*Ferritin:* Ferritin is decreased in iron deficiency anemia and increase in iron overload. It is measured with immunoassay with reference ranges of 20-380 ng/mL among men and 10-232 ng/mL among women.(Diagnostics)

|  |  |
| --- | --- |

*Erythrocyte Sedimentation Rate (ESR)*: Using 5 mL of refrigerated whole blood stored in lavender-top EDTA tubes, the ESR was tested within 24 hr of blood draw. This test used automated modified Westergren photochemical capillary stopped flow kinetic analysis.(Diagnostics;Larsson and Hansson, 2004) The Mayo clinic reports a reference of 0-22 mm/hr for men and 0-29 mm/hr for women(Mayo Clinic, 2017) and is considered a proxy measure for serum fibrinogen.(Yin et al., 2017)

*Serum iron:* 0.5-1 mL of fasting serum was collected, transported at room temperature (with heparin added) and refrigerated or frozen subsequently. Serum iron was measured with spectrophotometry, (Diagnostics;Samarina and Proskurnin, 2015) with reference ranges for men aged ≥30y set at 50-180 µg/dL and for women: 20-49y (40-190 µg /dL) and 50+y(45-160 µg /dL). (Diagnostics)

*MCV:* Also known as erythrocyte mean corpuscular volume, MCV is measured using standard electronic cell sizing/counting/cytometry/microscopy. Similar to other hemogram measures (e.g. ESR), a microtainer 1 mL whole blood in an EDTA (lavender-top) tube was transported at room temperature to the laboratory facility.(Diagnostics)

*MCH*: The hematologic index MCH was calculated as follows: MCH = Hb/RBC.

**B.** **Least absolute shrinkage and selection operator (LASSO) regression procedure**

In order to select the appropriate set of predictive model for each of the 3 vitamins, we used statistical learning method for variable selection known as adaptive LASSO, and compared it to cross-validation LASSO (cvLASSO) and lowest BIC LASSO. Socio-demographic variables, namely age, sex, race/ethnicity, poverty status were force entered in all models as fixed terms. The LASSO then selected among the other covariates listed above, the ones that should be retained. Covariates were imputed using chained equations (5 imputations, 10 iterations), accounting for their level of measurement. Socio-demographic factors were entered into all the chained equations. Continuous covariates were entered as outcomes in a series of linear regression models, while binary and categorical variables were entered into a series of multinomial logit regression models.

LASSO is a covariate selection methodology that is superior to both generalized linear models without covariate selection and the usually applied stepwise or backward elimination process.(Zou, 2006) In fact, stepwise selection is often trapped into a local optimal solution rather than the global optimal solution and backward elimination can be time-consuming given the large number of variables in the full model.(Zou, 2006) These methods often ignore stochastic errors or uncertainty incurred during variable selection, with the LASSO estimate being defined as follows:

β(lasso) = ${\arg min}_{\beta}$|| y – $\sum_{j=1}^{p} x_{j}\beta_{j}$ ||^2^ + $\lambda\sum_{j=1}^{p} |\beta_{j} |$

with $\lambda$ being a nonnegative regularization parameter.(Zou, 2006) The second term of the equation termed the “l1 penalty” is a key portion of this equation ensuring the success of the lasso method of covariate selection. In fact, this method was shown to discover the right sparse representation of the model, given certain conditions. Nevertheless, this method can produce biased estimates for larger coefficients. Thus, there a number of scenarios whereby the LASSO can yield inconsistent results. More recently, several related methods have been developed and validated against each other. It was shown that an adaptive version of the LASSO gave more consistent findings, particularly when compared with another popular variable selection technique known as the nonnegative garotte.

In our modeling approach, we used this convex optimization technique with l_1_ constraint known as adaptive LASSO as one of three methods to select the final linear regression models. The model is trained on a random half sample of the total population (first imputation out of 5) and validated against the other half sample to check robustness of findings, by comparing R^2^ between samples. One model was selected among the cvLASSO, adaptive LASSO or minBIC LASSO, depending on how close the R^2^ are between half-samples. This parsimonious model selected for each of 3 vitamins (measured at v_1_) as 6 potential outcomes is then run on the entire population and a backward elimination process is carried out to keep only significant covariates at type I error of 0.10. Thus, the selected model through LASSO was used as a starting point for further backward elimination. Backward elimination was conducted on the imputed data for the entire sample, rather than the half sample for the first imputation.

In our analysis, the following LASSO models were selected and the final model included is shown also in this Table.

|  |  | **Selected covariates^1^** | | | |
| --- | --- | --- | --- | --- | --- |
|  |  | **cvLASSO** | **Min BIC LASSO** | **Adaptive LASSO** | **Reduced model** |
|  |  |  |  |  |  |
| Vitamin D (v1) |  | Sex, race, pir, age,  B12, Folate, BMI, Cholesterol, ESR, MCV, Iron, Triglycerides, MAR, Albumin, education, Uric acid, MCH, NSAIDs, statins, Diabetes, WBC, CVD, HDL, RDW, education, current drug use, creatinine, DASH, Ferritin | **Sex, race, pir, age**  **B12, Folate, BMI, Cholesterol, MCV, MAR, Albumin** | Sex, race, pir, age,  B12, Folate, BMI, Cholesterol, ESR, MCV, Iron, Triglycerides, MAR, Albumin, education, Uric acid, NSAIDs, statins, Diabetes, WBC, CVD | **Sex, race, pir, age, B12, Folate, BMI, MCV, Albumin** |
| **Folate (v1)** |  | Age, sex, pir, race, B12, Vitamin D, MAR, Ferritin, iron, smoking, MCH, education, DASH, diagnosed diabetes, Albumin, CES-D, diagnosed hypertension, cholesterol, CRP, Hemoglobin, HbA1c, diagnosed dyslipidemia, RDW, NSAIDs, married. | **Age, sex, pir, race, B12, Vitamin D, MAR, Ferritin, iron, smoking, MCH, education, DASH, Diabetes, Albumin, CES-D, hypertension, cholesterol, CRP, Hemoglobin, HbA1c, diagnosed dyslipidemia, RDW, NSAIDs, married.** | Age, sex, pir, race, B12, Vitamin D, MAR, Ferritin, iron, smoking, MCH, education, DASH, Diabetes, Albumin, CES-D, hypertension, cholesterol, CRP, Hemoglobin, HbA1c, diagnosed dyslipidemia, RDW, NSAIDs. | **Age, sex, pir, race, B12, Vitamin D, MAR, Ferritin, iron, smoking, DASH, Albumin, Hemoglobin, RDW** |
| **B-12 (v1)** |  | Age, sex, race, pir, vitamin D, Folate, vitamin supplement use, HEI-2010, Ferritin, RDW, ESR, Triglycerides, MCH, Cholesterol, married albumin. | **Age, sex, race, pir, vitamin D, Folate, vitamin supplement use, HEI-2010, RDW.** | Age, sex, race, pir, vitamin D, Folate, vitamin supplement use, HEI-2010, Ferritin, RDW, ESR, Triglycerides, MCH, Cholesterol. | **Age, sex, race, pir, vitamin D, Folate, vitamin supplement use, HEI-2010, RDW.** |
|  |  |  |  |  |  |

*Abbreviations*: B-12=vitamin B-12 (cobalamin); BIC=Bayesian information criterion; BMI=Body Mass Index; CES-D=Center for Epidemiologic Studies-Depression; CRP=C-reactive Protein; cv=cross-validation; CVD=Self-reported cardiovascular disease; DASH=Dietary Approaches to Stop Hypertension; ESR=Erythrocyte Sedimentation Rate; HbA1c=Glycated hemoglobin; HDL=High Density Lipoprotein Cholesterol; LASSO= Least absolute shrinkage and selection operator; HEI-2010=Healthy Eating Index, 2010 revision; MAR=Mean Adequacy Ratio; MCH=Mean cell hemoglobin; MCV=Mean Cell Volume; NSAIDS=Non-Steroidal Anti-inflammatory Drugs; RDW=Red cell distribution Width; WBC=White Blood Cells; WHR=Waist-Hip-Ratio

^1^Bolded sets of covariates are the ones that are selected at each step of the model selection process. A full row of bolded sets of covariates indicates that the selection process is equivalent and that backward elimination did not reduce the model further.

**Supplemental Table 1**: BRAIN ATLAS NOMENCLATURE FOR sMRI data^1,2,3^

| **ROI_INDEX** | **NUM_VOX** | **TISSUE_SEG** | **HEMISPHERE** | **SUBGROUP_0** | **SUBGROUP_1** | **SUBGROUP_2** | **ROI_NAME** |
| --- | --- | --- | --- | --- | --- | --- | --- |
| 95 | 12872 | WM | B | CC |  |  | corpus callosum |
| 71 | 4899.8 | GM | B | CEREBELLUM |  |  | Cerebellar Vermal Lobules I-V |
| 73 | 2858.8 | GM | B |  |  |  | Cerebellar Vermal Lobules VIII-X |
| 72 | 2266.9 | GM | B |  |  |  | Cerebellar Vermal Lobules VI-VII |
| 39 | 54583 | GM | L |  |  |  | Left Cerebellum Exterior |
| 41 | 15501 | WM | L |  |  |  | Left Cerebellum White Matter |
| 38 | 54379 | GM | R |  |  |  | Right Cerebellum Exterior |
| 40 | 15459 | WM | R |  |  |  | Right Cerebellum White Matter |
| 30 | 585.9 | GM | L | DEEP_WM_GM | BASAL_GANGLIA |  | Left Accumbens Area |
| 37 | 3578.9 | GM | L |  |  |  | Left Caudate |
| 56 | 1597.6 | GM | L |  |  |  | Left Pallidum |
| 58 | 4942.3 | GM | L |  |  |  | Left Putamen |
| 23 | 526 | GM | R |  |  |  | Right Accumbens Area |
| 36 | 3651.5 | GM | R |  |  |  | Right Caudate |
| 55 | 1638.8 | GM | R |  |  |  | Right Pallidum |
| 57 | 4726 | GM | R |  |  |  | Right Putamen |
| 60 | 8574.1 | GM | L |  | DEEP_GM |  | Left Thalamus Proper |
| 59 | 8256.3 | GM | R |  |  |  | Right Thalamus Proper |
| 92 | 2887.7 | WM | L |  | DEEP_WM |  | anterior limb of internal capsule left |
| 91 | 3393.3 | WM | R |  |  |  | anterior limb of internal capsule right |
| 90 | 673.6 | WM | L |  |  |  | fornix left |
| 89 | 517.5 | WM | R |  |  |  | fornix right |
| 94 | 2416.3 | WM | L |  |  |  | posterior limb of internal capsule inc. cerebral peduncle left |
| 93 | 2480.5 | WM | R |  |  |  | posterior limb of internal capsule inc. cerebral peduncle right |
| 32 | 993.7 | GM | L |  |  |  | Left Amygdala |
| 75 | 586.5 | GM | L |  |  |  | Left Basal Forebrain |
| 48 | 3597.7 | GM | L |  |  |  | Left Hippocampus |
| 31 | 1021.3 | GM | R |  |  |  | Right Amygdala |
| 76 | 593.1 | GM | R |  |  |  | Right Basal Forebrain |
| 47 | 3704.7 | GM | R |  |  |  | Right Hippocampus |
| 105 | 1897.7 | GM | L | FRONTAL | FRONTAL_GM | FRONTAL_INFERIOR_GM | Left AOrG anterior orbital gyrus |
| 137 | 3015.9 | GM | L |  |  |  | Left LOrG lateral orbital gyrus |
| 147 | 4637.3 | GM | L |  |  |  | Left MOrG medial orbital gyrus |
| 179 | 2915.7 | GM | L |  |  |  | Left POrG posterior orbital gyrus |
| 104 | 2244.9 | GM | R |  |  |  | Right AOrG anterior orbital gyrus |
| 136 | 2864.1 | GM | R |  |  |  | Right LOrG lateral orbital gyrus |
| 146 | 4526.7 | GM |  |  |  |  | Right MOrG medial orbital gyrus |
| 178 | 2504.8 | GM | R |  |  |  | Right POrG posterior orbital gyrus |
| 103 | 4749.1 | GM | L |  |  | FRONTAL_INSULAR_GM | Left AIns anterior insula |
| 173 | 2479.5 | GM | L |  |  |  | Left PIns posterior insula |
| 102 | 4600.1 | GM | R |  |  |  | Right AIns anterior insula |
| 172 | 2532 | GM | R |  |  |  | Right PIns posterior insula |
| 121 | 4392.8 | GM | L |  |  | FRONTAL_LATERAL_GM | Left FRP frontal pole |
| 143 | 22847 | GM | L |  |  |  | Left MFG middle frontal gyrus |
| 163 | 3747 | GM | L |  |  |  | Left OpIFG opercular part of the inferior frontal gyrus |
| 165 | 1901.2 | GM | L |  |  |  | Left OrIFG orbital part of the inferior frontal gyrus |
| 183 | 14665 | GM | L |  |  |  | Left PrG precentral gyrus |
| 191 | 16867 | GM | L |  |  |  | Left SFG superior frontal gyrus |
| 205 | 5256.2 | GM | L |  |  |  | Left TrIFG triangular part of the inferior frontal gyrus |
| 120 | 4673.7 | GM | R |  |  |  | Right FRP frontal pole |
| 142 | 22580 | GM | R |  |  |  | Right MFG middle frontal gyrus |
| 162 | 4094.1 | GM | R |  |  |  | Right OpIFG opercular part of the inferior frontal gyrus |
| 164 | 1944.4 | GM | R |  |  |  | Right OrIFG orbital part of the inferior frontal gyrus |
| 182 | 14641 | GM | R |  |  |  | Right PrG precentral gyrus |
| 190 | 16697 | GM | R |  |  |  | Right SFG superior frontal gyrus |
| 204 | 4522.4 | GM | R |  |  |  | Right TrIFG triangular part of the inferior frontal gyrus |
| 125 | 2920.3 | GM | L |  |  | FRONTAL_MEDIAL_GM | Left GRe gyrus rectus |
| 141 | 2245.2 | GM | L |  |  |  | Left MFC medial frontal cortex |
| 151 | 3081.3 | GM | L |  |  |  | Left MPrG precentral gyrus medial segment |
| 153 | 8737 | GM | L |  |  |  | Left MSFG superior frontal gyrus medial segment |
| 187 | 1220.8 | GM | L |  |  |  | Left SCA subcallosal area |
| 193 | 6723.3 | GM | L |  |  |  | Left SMC supplementary motor cortex |
| 124 | 2699.9 | GM | R |  |  |  | Right GRe gyrus rectus |
| 140 | 2202.6 | GM | R |  |  |  | Right MFC medial frontal cortex |
| 150 | 2944.5 | GM | R |  |  |  | Right MPrG precentral gyrus medial segment |
| 152 | 9415.8 | GM | R |  |  |  | Right MSFG superior frontal gyrus medial segment |
| 186 | 1236 | GM | R |  |  |  | Right SCA subcallosal area |
| 192 | 6368.8 | GM | R |  |  |  | Right SMC supplementary motor cortex |
| 113 | 4466.1 | GM | L |  |  | FRONTAL_OPERCULAR_GM | Left CO central operculum |
| 119 | 2489.9 | GM | L |  |  |  | Left FO frontal operculum |
| 175 | 2768.9 | GM | L |  |  |  | Left PO parietal operculum |
| 112 | 4691.3 | GM | R |  |  |  | Right CO central operculum |
| 118 | 2548.3 | GM | R |  |  |  | Right FO frontal operculum |
| 174 | 2414.5 | GM | R |  |  |  | Right PO parietal operculum |
| 82 | 91872 | WM | L |  | FRONTAL_WM |  | frontal lobe WM left |
| 81 | 95088 | WM | R |  |  |  | frontal lobe WM right |
| 101 | 5262.2 | GM | L | LIMBIC | LIMBIC_GM | LIMBIC_CINGULATE_GM | Left ACgG anterior cingulate gyrus |
| 139 | 5335.1 | GM | L |  |  |  | Left MCgG middle cingulate gyrus |
| 167 | 5181.6 | GM | L |  |  |  | Left PCgG posterior cingulate gyrus |
| 100 | 4782.3 | GM | R |  |  |  | Right ACgG anterior cingulate gyrus |
| 138 | 5475.1 | GM | R |  |  |  | Right MCgG middle cingulate gyrus |
| 166 | 4324.3 | GM | R |  |  |  | Right PCgG posterior cingulate gyrus |
| 117 | 1887.4 | GM | L |  |  | LIMBIC_MEDIALTEMPORAL_GM | Left Ent entorhinal area |
| 171 | 3536.5 | GM | L |  |  |  | Left PHG parahippocampal gyrus |
| 116 | 2120.6 | GM | R |  |  |  | Right Ent entorhinal area |
| 170 | 3257.5 | GM | R |  |  |  | Right PHG parahippocampal gyrus |
| 161 | 5087.5 | GM | L | OCCIPITAL | OCCIPITAL_GM | OCCIPITAL_INFERIOR_GM | Left OFuG occipital fusiform gyrus |
| 160 | 4857.3 | GM | R |  |  |  | Right OFuG occipital fusiform gyrus |
| 129 | 7403.4 | GM | L |  |  | OCCIPITAL_LATERAL_GM | Left IOG inferior occipital gyrus |
| 145 | 7232.9 | GM | L |  |  |  | Left MOG middle occipital gyrus |
| 157 | 4297.6 | GM | L |  |  |  | Left OCP occipital pole |
| 197 | 4152 | GM | L |  |  |  | Left SOG superior occipital gyrus |
| 128 | 7633 | GM | R |  |  |  | Right IOG inferior occipital gyrus |
| 144 | 6792.1 | GM | R |  |  |  | Right MOG middle occipital gyrus |
| 156 | 4054.5 | GM | R |  |  |  | Right OCP occipital pole |
| 196 | 4967 | GM | R |  |  |  | Right SOG superior occipital gyrus |
| 109 | 3635.5 | GM | L |  |  | OCCIPITAL_MEDIAL_GM | Left Calc calcarine cortex |
| 115 | 5314.7 | GM | L |  |  |  | Left Cun cuneus |
| 135 | 8386.3 | GM | L |  |  |  | Left LiG lingual gyrus |
| 108 | 3543.7 | GM | R |  |  |  | Right Calc calcarine cortex |
| 114 | 5884.9 | GM | R |  |  |  | Right Cun cuneus |
| 134 | 8366 | GM | R |  |  |  | Right LiG lingual gyrus |
| 84 | 22742 | WM | L |  | OCCIPITAL_WM |  | occipital lobe WM left |
| 83 | 22799 | WM | R |  |  |  | occipital lobe WM right |
| 107 | 9939.4 | GM | L | PARIETAL | PARIETAL_GM | PARIETAL_LATERAL_GM | Left AnG angular gyrus |
| 177 | 13594 | GM | L |  |  |  | Left PoG postcentral gyrus |
| 195 | 9984.3 | GM | L |  |  |  | Left SMG supramarginal gyrus |
| 199 | 11733 | GM | L |  |  |  | Left SPL superior parietal lobule |
| 106 | 11564 | GM | R |  |  |  | Right AnG angular gyrus |
| 176 | 11681 | GM | R |  |  |  | Right PoG postcentral gyrus |
| 194 | 9193 | GM | R |  |  |  | Right SMG supramarginal gyrus |
| 198 | 11792 | GM | R |  |  |  | Right SPL superior parietal lobule |
| 149 | 1400.3 | GM | L |  |  | PARIETAL_MEDIAL_GM | Left MPoG postcentral gyrus medial segment |
| 169 | 11737 | GM | L |  |  |  | Left PCu precuneus |
| 148 | 1162.5 | GM | R |  |  |  | Right MPoG postcentral gyrus medial segment |
| 168 | 11732 | GM | R |  |  |  | Right PCu precuneus |
| 86 | 47237 | WM | L |  | PARIETAL_WM |  | parietal lobe WM left |
| 85 | 44217 | WM | R |  |  |  | parietal lobe WM right |
| 123 | 8077.2 | GM | L | TEMPORAL | TEMPORAL_GM | TEMPORAL_INFERIOR_GM | Left FuG fusiform gyrus |
| 122 | 8000.9 | GM | R |  |  |  | Right FuG fusiform gyrus |
| 133 | 12612 | GM | L |  |  | TEMPORAL_LATERAL_GM | Left ITG inferior temporal gyrus |
| 155 | 15794 | GM | L |  |  |  | Left MTG middle temporal gyrus |
| 201 | 8451.3 | GM | L |  |  |  | Left STG superior temporal gyrus |
| 203 | 8632.1 | GM | L |  |  |  | Left TMP temporal pole |
| 132 | 12693 | GM | R |  |  |  | Right ITG inferior temporal gyrus |
| 154 | 16085 | GM | R |  |  |  | Right MTG middle temporal gyrus |
| 200 | 9031.2 | GM | R |  |  |  | Right STG superior temporal gyrus |
| 202 | 8883.7 | GM | R |  |  |  | Right TMP temporal pole |
| 181 | 2629.8 | GM | L |  |  | TEMPORAL_SUPRATEMPORAL_GM | Left PP planum polare |
| 185 | 2511.3 | GM | L |  |  |  | Left PT planum temporale |
| 207 | 1821.1 | GM | L |  |  |  | Left TTG transverse temporal gyrus |
| 180 | 2448.5 | GM | R |  |  |  | Right PP planum polare |
| 184 | 2325.5 | GM | R |  |  |  | Right PT planum temporale |
| 206 | 1529.1 | GM | R |  |  |  | Right TTG transverse temporal gyrus |
| 88 | 54535 | WM | L |  | TEMPORAL_WM |  | temporal lobe WM left |
| 87 | 55391 | WM | R |  |  |  | temporal lobe WM right |
| 4 | 636.8 | VN | B | VENTRICLE |  |  | 3rd Ventricle |
| 11 | 1959.6 | VN | B |  |  |  | 4th Ventricle |
| 50 | 304.9 | VN | L |  |  |  | Left Inf Lat Vent |
| 52 | 7954.9 | VN | L |  |  |  | Left Lateral Ventricle |
| 49 | 352.9 | VN | R |  |  |  | Right Inf Lat Vent |
| 51 | 6629.5 | VN | R |  |  |  | Right Lateral Ventricle |
| 35 | 18492 | NONE | B |  |  |  | Brain Stem |
| 46 | 1011.6 | CSF | B |  |  |  | CSF |
| 62 | 5192.8 | NONE | L |  |  |  | Left Ventral DC |
| 64 | 36.5 | NONE | L |  |  |  | Left vessel |
| 61 | 4998.9 | NONE | R |  |  |  | Right Ventral DC |
| 63 | 33.3 | NONE | R |  |  |  | Right vessel |

^1^Shaded in light orange: Analysis A which consisted of TOTALBRAIN, WM and GM as alternative outcomes.

^2^Shaded in light green: Analysis B which consisted of GM//WM categorized by larger regions: OCCIPITAL, PARIETAL, TEMPORAL and FRONTAL. This analysis included R and L summed together for each large region.

^3^Shaded in light gray: Analysis C which consisted of all available smaller regions. Excluded regions due to missing data are the ones in the last column that are not highlighted in gray. Additional regions included: Optic chiasm, Lesion Volume.

**Supplemental Table 2**: Regions of Interest (ROI) used for dMRI measures: Fractional anisotropy (FA) and trace (TR)^1^

| **LEFT BRAIN** | | |  |
| --- | --- | --- | --- |
| **1** | **SPG_L** | **Superior Parietal Gyrus Left** | GM |
| **2** | **CingG_L** | **Cingulate Gyrus Left** | GM |
| **3** | **SFG_L** | **Superior Frontal Gyrus Left** | GM |
| **4** | **MFG_L** | **Middle Frontal Gyrus Left** | GM |
| **5** | **IFG_L** | **Inferior Frontal Gyrus Left** | GM |
| **6** | **PrCG_L** | **Precentral Gyrus Left** | GM |
| **7** | **PoCG_L** | **Postcentral Gyrus Left** | GM |
| **8** | **AG_L** | **Angular Gyrus Left** | GM |
| **9** | **PrCu_L** | **Pre-Cuneus Left** | GM |
| **10** | **Cu_L** | **Cuneus Left** | GM |
| **11** | **LG_L** | **Lingual Gyrus Left** | GM |
| **12** | **Fu_L** | **Fusiform Gyrus Left** | GM |
| **13** | **PHG_L** | **Parahippocampal Gyrus Left** | GM |
| **14** | **SOG_L** | **Superior Occipital Gyrus Left** | GM |
| **15** | **IOG_L** | **Inferior Occipital Gyrus** | GM |
| **16** | **MOG_L** | **Middle Occipital Gyrus** | GM |
| **17** | **ENT_L** | **Entorhinal Area** | GM |
| **18** | **STG_L** | **Superior Temporal Gyrus** | GM |
| **19** | **ITG_L** | **Inferior Temporal Gyrus** | GM |
| **20** | **MTG_L** | **Middle Temporal Gyrus** | GM |
| **21** | **LFOG_L** | **Lateral Fronto-Orbital Gyrus** | GM |
| **22** | **MFOG_L** | **Middle Fronto-Orbital Gyrus** | GM |
| **23** | **SMG_L** | **Supramarginal Gyrus** | GM |
| **24** | **RG_L** | **Gyrus Rectus** | GM |
| **25** | **Ins_L** | **Insular** | GM |
| **26** | **Amyg_L** | **Amygdala** | GM |
| **27** | **Hippo_L** | **Hippocampus** | GM |
| **28** | **Cerebrellum_L** | **Cerebellum** | GM |
| **29** | **CST_L** | **Corticospinal Tract Left** | **WM** |
| **30** | **ICP_L** | **Inferior Cerebellar Peduncle Left** | **WM** |
| **31** | **ML_L** | **Medial Lemniscus Left** | **WM/GM** |
| **32** | **SCP_L** | **Superior Cerebellar Peduncle Left** | **WM** |
| **33** | **CP_L** | **Cerebellar Peduncle Left** | **WM** |
| **34** | **ALIC_L** | **Anterior Limb of Internal Capsule Left** | **WM** |
| **35** | **PLIC_L** | **Posterior Limb of Internal Capsule Left** | **WM** |
| **36** | **PTR_L** | **Posterior Thalamic Radiation (Include Optic Radiation) Left** | **WM** |
| **37** | **ACR_L** | **Anterior Corona Radiata Left** | **WM** |
| **38** | **SCR_L** | **Superior Corona Radiata Left** | **WM** |
| **39** | **PCR_L** | **Posterior Corona Radiata Left** | **WM** |
| **40** | **CGC_L** | **Cingulum (Cingulate Gyrus) Left** | **WM** |
| **41** | **CGH_L** | **Cingulum (Hippocampus) Left** | **WM** |
| **42** | **Fx/ST_L** | **Fornix (Cres) / Stria Terminalis (Can Not Be Resolved With Current Resolution) Left** | **WM** |
| **43** | **SLF_L** | **Superior Longitudinal Fasciculus Left** | **WM** |
| **44** | **SFO_L** | **Superior Fronto-Occipital Fasciculus (Could Be A Part of Anterior Internal Capsule) Left** | **WM** |
| **45** | **IFO_L** | **Inferior Fronto-Occipital Fasciculus Left** | **WM** |
| **46** | **SS_L** | **Sagittal Stratum (Include Inferior Longitidinal Fasciculus And Inferior Fronto-Occipital**  **Fasciculus) Left** | **WM** |
| **47** | **EC_L** | **External Capsule Left** | **WM** |
| **48** | **UNC_L** | **Uncinate Fasciculus Left** | **WM** |
| **49** | **PCT_L** | **Pontine Crossing Tract (A Part of Mcp) Left** | **WM** |
| **50** | **MCP_L** | **Middle Cerebellar Peduncle Left** | **WM** |
| **51** | **FX_L** | **Fornix (Column And Body of Fornix) Left** | **WM** |
| **52** | **GCC_L** | **Genu of Corpus Callosum Left** | **WM** |
| **53** | **BCC_L** | **Body of Corpus Callosum Left** | **WM** |
| **54** | **SCC_L** | **Splenium of Corpus Callosum Left** | **WM** |
| **55** | **RLIC_L** | **Retrolenticular Part of Internal Capsule Left** | **WM** |
| **56** | **REDNC_L** | **Red Nucleus Left** | GM |
| **57** | **SNIGRA_L** | **Substancia Nigra Left** | GM |
| **58** | **TAP_L** | **Tapatum Left** | GM |
| **59** | **Caud_L** | **Caudate Nucleus Left** | GM |
| **60** | **Put_L** | **Putamen Left** | GM |
| **61** | **Thal_L** | **Thalamus Left** | GM |
| **62** | **GP_L** | **Globus Pallidus Left** | GM |
| **63** | **Midbrain_L** | **Midbrain Left** | GM |
| **64** | **Pons_L** | **Pons Left** | **WM** |
| **65** | **Medulla_L** | **Medulla Left** | **WM/GM** |
| **66** | **SPWM_L** | **Superior Parietal WM Left** | **WM** |
| **67** | **Cingwm** | **Cingulum WM Left** | **WM** |
| **68** | **SFWM_L** | **Superior Frontal WM Left** | **WM** |
| **69** | **MFWM_L** | **Middle Frontal WM Left** | **WM** |
| **70** | **IFWM_L** | **Inferior Frontal WM Left** | **WM** |
| **71** | **PrCWM_L** | **Precentral WM Left** | **WM** |
| **72** | **PoCWM_L** | **Postcentral WM Left** | **WM** |
| **73** | **AWM_L** | **Angular WM Left** | **WM** |
| **74** | **PrCuWM_L** | **Pre-Cuneus WM Left** | **WM** |
| **75** | **CuWM_L** | **Cuneus WM Left** | **WM** |
| **76** | **LWM_L** | **Lingual WM Left** | **WM** |
| **77** | **Fu_WM_L** | **Fusiform WM Left** | **WM** |
| **78** | **SOWM_L** | **Superior Occipital WM Left** | **WM** |
| **79** | **IOWM_L** | **Inferior Occipital WM Left** | **WM** |
| **80** | **MOWM_L** | **Middle Occipital WM Left** | **WM** |
| **81** | **STwm_L** | **Superior Temporal WM Left** | **WM** |
| **82** | **ITWM_L** | **Inferior Temporal WM Left** | **WM** |
| **83** | **MTWM_L** | **Middle Temporal WM Left** | **WM** |
| **84** | **LFOWM_L** | **Lateral Fronto-Orbital WM Left** | **WM** |
| **85** | **MFOWM_L** | **Middle Fronto-Orbital WM Left** | **WM** |
| **86** | **SMWM_L** | **Supramarginal WM Left** | **WM** |
| **87** | **RGWM_L** | **Rectus WM Left** | **WM** |
| **88** | **Cerebrellumwm_L** | **Cerebellum WM Left** | **WM** |
| **RIGHT BRAIN** | | | |
| **89** | **SPG_R** | **Superior Parietal Gyrus Right** | GM |
| **90** | **CingG_R** | **Cingulate Gyrus Right** | GM |
| **91** | **SFG_R** | **Superior Frontal Gyrus Right** | GM |
| **92** | **MFG_R** | **Middle Frontal Gyrus Right** | GM |
| **93** | **IFG_R** | **Inferior Frontal Gyrus Right** | GM |
| **94** | **PrCG_R** | **Precentral Gyrus Right** | **GM** |
| **95** | **PoCG_R** | **Postcentral Gyrus Right** | **GM** |
| **96** | **AG_R** | **Angular Gyrus Right** | **GM** |
| **97** | **PrCu_R** | **Pre-Cuneus Right** | **GM** |
| **98** | **Cu_R** | **Cuneus Right** | **GM** |
| **99** | **LG_R** | **Lingual Gyrus Right** | **GM** |
| **100** | **FuG_R** | **Fusiform Gyrus Right** | **GM** |
| **101** | **PHG_R** | **Parahippocampal Gyrus Right** | **GM** |
| **102** | **SOG_R** | **Superior Occipital Gyrus Right** | **GM** |
| **103** | **IOG_R** | **Inferior Occipital Gyrus Right** | **GM** |
| **104** | **MOG_R** | **Middle Occipital Gyrus Right** | **GM** |
| **105** | **ENT_R** | **Entorhinal Area Right** | **GM** |
| **106** | **STG_R** | **Superior Temporal Gyrus Right** | **GM** |
| **107** | **ITG_R** | **Inferior Temporal Gyrus Right** | **GM** |
| **108** | **MTG_R** | **Middle Temporal Gyrus Right** | **GM** |
| **109** | **LFOG_R** | **Lateral Fronto-Orbital Gyrus Right** | **GM** |
| **110** | **MFOG_R** | **Middle Fronto-Orbital Gyrus Right** | **GM** |
| **111** | **SMG_R** | **Supramarginal Gyrus Right** | **GM** |
| **112** | **RG_R** | **Gyrus Rectus Right** | **GM** |
| **113** | **Ins_R** | **Insular Right** | **GM** |
| **114** | **Amyg_R** | **Amygdala Right** | **GM** |
| **115** | **Hippo_R** | **Hippocampus Right** | **GM** |
| **116** | **Cerebellum_R** | **Cerebellum Right** | **GM** |
| **117** | **CST_R** | **Corticospinal Tract Right** | **WM** |
| **118** | **ICP_R** | **Inferior Cerebellar Peduncle Right** | **WM** |
| **119** | **ML_R** | **Medial Lemniscus Right** | **WM/GM** |
| **120** | **SCP_R** | **Superior Cerebellar Peduncle Right** | **WM** |
| **121** | **CP_R** | **Cerebellar peduncle, Right** |  |
| **122** | **ALIC_R** | **Anterior Limb of Internal Capsule Right** | **WM** |
| **123** | **PLIC_R** | **Posterior Limb of Internal Capsule Right** | **WM** |
| **124** | **PTR_R** | **Posterior Thalamic Radiation (Include Optic Radiation) Right** | **WM** |
| **125** | **ACR_R** | **Anterior Corona Radiata Right** | **WM** |
| **126** | **SCR_R** | **Superior Corona Radiata Right** | **WM** |
| **127** | **PCR_R** | **Posterior Corona Radiata Right** | **WM** |
| **128** | **CGC_R** | **Cingulum (Cingulate Gyrus) Right** | **WM** |
| **129** | **CGH_R** | **Cingulum (Hippocampus) Right** | **WM** |
| **130** | **Fx/ST_R** | **Fornix (Cres) / Stria Terminalis (Can Not Be Resolved With Current Resolution) Right** | **WM** |
| **131** | **SLF_R** | **Superior Longitudinal Fasciculus Right** | **WM** |
| **132** | **SFO_R** | **Superior Fronto-Occipital Fasciculus (Could Be A Part of Anterior Internal Capsule) Right** | **WM** |
| **133** | **IFO_R** | **Inferior Fronto-Occipital Fasciculus Right** | **WM** |
| **134** | **SS_R** | **Sagittal Stratum (Include Inferior Longitidinal Fasciculus And Inferior Fronto-Occipital Fasciculus) Right** | **WM** |
| **135** | **EC_R** | **External Capsule Right** | **WM** |
| **136** | **UNC_R** | **Uncinate Fasciculus Right** | **WM** |
| **137** | **PCT_R** | **Pontine Crossing Tract (A Part of MCP) Right** | **WM** |
| **138** | **MCP_R** | **Middle Cerebellar Peduncle Right** | **WM** |
| **139** | **FX_R** | **Fornix (Column And Body of Fornix) Right** | **WM** |
| **140** | **GCC_R** | **Genu of Corpus Callosum Right** | **WM** |
| **141** | **BCC_R** | **Body of Corpus Callosum Right** | **WM** |
| **142** | **SCC_R** | **Splenium of Corpus Callosum Right** | **WM** |
| **143** | **RLIC_R** | **Retrolenticular Part of Internal Capsule Right** | **WM** |
| **144** | **REDNC_R** | **Red Nucleus Right** | **GM** |
| **145** | **SNIGRA_R** | **Substancia Nigra Right** | **GM** |
| **146** | **TAP_R** | **Tapatum Right** | **GM** |
| **147** | **Caud_R** | **Caudate Nucleus Right** | **GM** |
| **148** | **Put_R** | **Putamen Right** | **GM** |
| **149** | **Thal_R** | **Thalamus Right** | **GM** |
| **150** | **GP_R** | **Globus Pallidus Right** | **GM** |
| **151** | **Midbrain_R** | **Midbrain Right** | **GM** |
| **152** | **Pons_R** | **Pons Right** | **WM** |
| **153** | **Medulla_R** | **Medulla Right** | **WM/GM** |
| **154** | **SPwm_R** | **Superior Parietal WM Right** | **WM** |
| **155** | **Cingwm_R** | **Cingulum WM Right** | **WM** |
| **156** | **SFWM_R** | **Superior Frontal WM Right** | **WM** |
| **157** | **MFWM_R** | **Middle Frontal WM Right** | **WM** |
| **158** | **IFWM_R** | **Inferior Frontal WM Right** | **WM** |
| **159** | **PrCWM_R** | **Precentral WM Right** | **WM** |
| **160** | **PoCWM_R** | **Postcentral WM Right** | **WM** |
| **161** | **AWM_R** | **Angular WM Right** | **WM** |
| **162** | **PrCuWM_R** | **Pre-Cuneus WM Right** | **WM** |
| **163** | **CuWM_R** | **Cuneus WM Right** | **WM** |
| **164** | **LWM_R** | **Lingual WM Right** | **WM** |
| **165** | **Fuwm_R** | **Fusiform WM Right** | **WM** |
| **166** | **SOWM_R** | **Superior Occipital WM Right** | **WM** |
| **167** | **IOWM_R** | **Inferior Occipital WM Right** | **WM** |
| **168** | **MOWM_R** | **Middle Occipital WM Right** | **WM** |
| **169** | **STWM_R** | **Superior Temporal WM Right** | **WM** |
| **170** | **ITWM_R** | **Inferior Temporal WM Right** | **WM** |
| **171** | **MTWM_R** | **Middle Temporal WM Right** | **WM** |
| **172** | **LFOWM_R** | **Lateral Fronto-Orbital WM Right** | **WM** |
| **173** | **MFOWM_R** | **Middle Fronto-Orbital WM Right** | **WM** |
| **174** | **SMWM_R** | **Supramarginal WM Right** | **WM** |
| **175** | **RGWM_R** | **Rectus WM Right** | **WM** |
| **176** | **Cerebrellumwm_R** | **Cerebellum WM Right** | **WM** |

^1^Right and Left measures of FA and TR were averaged out before analyses C and D was carried out. This resulted in 98 measures in total, 49 for FA and 49 for TR, when excluding measures with missing data. Measures included in the analysis are bolded and in red font. All others are excluded. In addition, cerebellum wm TR (Right and Left) were only available for 85 subjects, as was the case for SNIGRA FA/TR (Right and Left). TR is also known as mean diffusivity or MD.

**Supplemental References**:

Andersson, J.L.R., and Sotiropoulos, S.N. (2016). An integrated approach to correction for off-resonance effects and subject movement in diffusion MR imaging. *Neuroimage* 125**,** 1063-1078.

Beydoun, M.A., Beydoun, H.A., Mode, N., Dore, G.A., Canas, J.A., Eid, S.M., and Zonderman, A.B. (2016a). Racial disparities in adult all-cause and cause-specific mortality among us adults: mediating and moderating factors. *BMC Public Health* 16**,** 1113.

Beydoun, M.A., Canas, J.A., Dore, G.A., Beydoun, H.A., Rostant, O.S., Fanelli-Kuczmarski, M.T., Evans, M.K., and Zonderman, A.B. (2016b). Serum Uric Acid and Its Association with Longitudinal Cognitive Change Among Urban Adults. *J Alzheimers Dis* 52**,** 1415-1430.

Beydoun, M.A., Hossain, S., Fanelli-Kuczmarski, M.T., Beydoun, H.A., Canas, J.A., Evans, M.K., and Zonderman, A.B. (2018). Vitamin D Status and Intakes and Their Association With Cognitive Trajectory in a Longitudinal Study of Urban Adults. *J Clin Endocrinol Metab* 103**,** 1654-1668.

Beydoun, M.A., Obhi, H.K., Weiss, J., Canas, J.A., Beydoun, H.A., Evans, M.K., and Zonderman, A.B. (2019). Systemic inflammation is associated with depressive symptoms differentially by sex and race: a longitudinal study of urban adults. *Mol Psychiatry*.

Centers for Disease Control and Prevention (2007). *National Health and Nutrition Examination Surveys 2007-2008:* [*https://wwwn.cdc.gov/nchs/nhanes/continuousnhanes/default.aspx?BeginYear=2007*](https://wwwn.cdc.gov/nchs/nhanes/continuousnhanes/default.aspx?BeginYear=2007) [Online]. Available: <https://wwwn.cdc.gov/nchs/nhanes/continuousnhanes/default.aspx?BeginYear=2007> [Accessed].

Diagnostics, Q. *Ferritin* [Online]. Available: <https://www.questdiagnostics.com/testcenter/TestDetail.action?ntc=457&searchString=8272> [Accessed May 13sth 2019].

Diagnostics, Q. *Hemogram* [Online]. Available: <https://www.questdiagnostics.com/testcenter/BUOrderInfo.action?tc=7008&labCode=DAL> [Accessed May 13sth 2019].

Diagnostics, Q. *Iron, Total and Total Iron Binding Capacity* [Online]. Available: <https://www.questdiagnostics.com/testcenter/BUOrderInfo.action?tc=7573&labCode=SEA> [Accessed May 13sth 2019].

Doshi, J., Erus, G., Ou, Y., Gaonkar, B., and Davatzikos, C. (2013). Multi-atlas skull-stripping. *Acad Radiol* 20**,** 1566-1576.

Doshi, J., Erus, G., Ou, Y., Resnick, S.M., Gur, R.C., Gur, R.E., Satterthwaite, T.D., Furth, S., Davatzikos, C., and Alzheimer's Neuroimaging, I. (2016). MUSE: MUlti-atlas region Segmentation utilizing Ensembles of registration algorithms and parameters, and locally optimal atlas selection. *Neuroimage* 127**,** 186-195.

Fanelli Kuczmarski, M., Bodt, B.A., Stave Shupe, E., Zonderman, A.B., and Evans, M.K. (2018). Dietary Patterns Associated with Lower 10-Year Atherosclerotic Cardiovascular Disease Risk among Urban African-American and White Adults Consuming Western Diets. *Nutrients* 10.

Fanelli Kuczmarski, M., Mason, M.A., Beydoun, M.A., Allegro, D., Zonderman, A.B., and Evans, M.K. (2013). Dietary patterns and sarcopenia in an urban African American and White population in the United States. *J Nutr Gerontol Geriatr* 32**,** 291-316.

Gunter, J.L., Bernstein, M.A., Borowski, B.J., Ward, C.P., Britson, P.J., Felmlee, J.P., Schuff, N., Weiner, M., and Jack, C.R. (2009). Measurement of MRI scanner performance with the ADNI phantom. *Med Phys* 36**,** 2193-2205.

Hisamatsu, T., Fujiyoshi, A., Miura, K., Ohkubo, T., Kadota, A., Kadowaki, S., Kadowaki, T., Yamamoto, T., Miyagawa, N., Zaid, M., Torii, S., Takashima, N., Murakami, Y., Okamura, T., Horie, M., Ueshima, H., and Group, S.R. (2014). Lipoprotein particle profiles compared with standard lipids in association with coronary artery calcification in the general Japanese population. *Atherosclerosis* 236**,** 237-243.

Jones, D.K. (2008). Studying connections in the living human brain with diffusion MRI. *Cortex* 44**,** 936-952.

Larsson, A., and Hansson, L.O. (2004). Analysis of inflammatory response in human plasma samples by an automated multicapillary electrophoresis system. *Clin Chem Lab Med* 42**,** 1396-1400.

Li, C., Gore, J.C., and Davatzikos, C. (2014). Multiplicative intrinsic component optimization (MICO) for MRI bias field estimation and tissue segmentation. *Magn Reson Imaging* 32**,** 913-923.

Manickam, P., Rathod, A., Panaich, S., Hari, P., Veeranna, V., Badheka, A., Jacob, S., and Afonso, L. (2011). Comparative prognostic utility of conventional and novel lipid parameters for cardiovascular disease risk prediction: do novel lipid parameters offer an advantage? *J Clin Lipidol* 5**,** 82-90.

Mayo Clinic (2017). *Sed rate (erythrocyte sedimentation rate)* [Online]. Rochester, MN. Available: <https://www.mayoclinic.org/tests-procedures/sed-rate/about/pac-20384797> [Accessed May 10th 2019].

Mellen, P.B., Gao Sk Fau - Vitolins, M.Z., Vitolins Mz Fau - Goff, D.C., Jr., and Goff, D.C., Jr. Deteriorating dietary habits among adults with hypertension: DASH dietary accordance, NHANES 1988-1994 and 1999-2004.

Mulkern, R.V., Forbes, P., Dewey, K., Osganian, S., Clark, M., Wong, S., Ramamurthy, U., Kun, L., and Poussaint, T.Y. (2008). Establishment and results of a magnetic resonance quality assurance program for the pediatric brain tumor consortium. *Acad Radiol* 15**,** 1099-1110.

Murakami, K., Livingstone, M.B.E., and Sasaki, S. (2019). Diet quality scores in relation to metabolic risk factors in Japanese adults: a cross-sectional analysis from the 2012 National Health and Nutrition Survey, Japan. *Eur J Nutr* 58**,** 2037-2050.

Murphy, S.P., Foote, J.A., Wilkens, L.R., Basiotis, P.P., Carlson, A., White, K.K., and Yonemori, K.M. (2006). Simple measures of dietary variety are associated with improved dietary quality. *J Am Diet Assoc* 106**,** 425-429.

Myers, J.K., and Weissman, M.M. (1980). Use of a self-report symptom scale to detect depression in a community sample. *Am J Psychiatry* 137**,** 1081-1084.

Nair, D., Carrigan, T.P., Curtin, R.J., Popovic, Z.B., Kuzmiak, S., Schoenhagen, P., Flamm, S.D., and Desai, M.Y. (2009). Association of total cholesterol/ high-density lipoprotein cholesterol ratio with proximal coronary atherosclerosis detected by multislice computed tomography. *Prev Cardiol* 12**,** 19-26.

Nguyen, H.T., Kitner-Triolo, M., Evans, M.K., and Zonderman, A.B. (2004). Factorial invariance of the CES-D in low socioeconomic status African Americans compared with a nationally representative sample. *Psychiatry Res* 126**,** 177-187.

Phantom, N.D.).

Radloff, L. (1977). The CES-D scale: a self-report depression scale for research in the general population. *Applied Psychological Measurement* 1.

Ramos, M.I., Allen, L.H., Haan, M.N., Green, R., and Miller, J.W. (2004). Plasma folate concentrations are associated with depressive symptoms in elderly Latina women despite folic acid fortification. *Am J Clin Nutr* 80**,** 1024-1028.

Samarina, T., and Proskurnin, M. (2015). Rapid assessment of iron in blood plasma and serum by spectrophotometry with cloud-point extraction. *F1000Res* 4**,** 623.

Techs, O.L.C.E.F.C.L.a.M. (2019). Red Blood Cell Distribution Width (RDW): Definition and Calculation.

Tristan-Vega, A., and Aja-Fernandez, S. (2010). DWI filtering using joint information for DTI and HARDI. *Med Image Anal* 14**,** 205-218.

Yin, W., Xu, Z., Sheng, J., Xie, X., and Zhang, C. (2017). Erythrocyte sedimentation rate and fibrinogen concentration of whole blood influences the cellular composition of platelet-rich plasma obtained from centrifugation methods. *Exp Ther Med* 14**,** 1909-1918.

Zou, H. (2006). The adaptive Lasso and it oracle properties. *Journal of the American Statistical Association* 101**,** 1418-1428.
